# A Novel Web‐Based Approach for Monitoring Biodiversity

**DOI:** 10.1002/ece3.70364

**Published:** 2024-09-29

**Authors:** Rajendra Mohan Panda, Padmanava Dash, Partha Sarathi Roy

**Affiliations:** ^1^ Department of Integrative Biology University of South Florida Tampa Florida USA; ^2^ Department of Geosciences Mississippi State University Starkville Mississippi USA; ^3^ Sustainable Landscapes and Restoration World Resources Institute India New Delhi India

**Keywords:** biodiversity conservation, ecosystem modeling, interactive visualization, multicolinearity, species‐environment relationships

## Abstract

Understanding complexities in biodiversity is one of the fundamental goals of ecology and its monitoring is significant for ecosystem sustainability, maintenance, and conservation. However, biodiversity monitoring needs improvement to handle complex datasets and their analyses. This study attempts to understand these ecological complexities quickly, efficiently, and easily. The aim is to provide an alternative to ecologists, researchers, instructors, and stakeholders for biodiversity monitoring with the flexibility to visualize and customize outputs without software knowledge. A novel web‐based technique is applied to monitor the biodiversity of a complex mountain ecosystem using a national database. The species–environment relationships of different vegetation types across a mountain ecosystem's elevation gradient are investigated using open‐source climatic, physiographic, and socioeconomic variables. The proposed interactive tool to monitor biodiversity and understand its complexities is designed to visualize the data structure, summary, correlations, and sampling effectiveness quickly and easily. Plant species richness patterns and life forms (herb, shrub, and tree) across elevational gradients are investigated. We highlight the preliminary investigation of the data structure and their spatial distribution and apply the multicollinearity test to select variables for modeling. The drop‐down menu helps users browse different datasets and select those datasets for instant visualization. Preliminary investigations on interactions between variables and species richness of vegetation types along elevation gradient interactively displayed with options to select variables, plant richness, and an elevational range. Species–environment relationships are investigated using multiple modeling protocols, and results are interactively displayed with options to download in different file formats and colors at the click of a button. This visualization tool helps to understand ecosystem structure, species richness patterns and species–environment relationships easily and efficiently. The R‐codes used in this tool are reproducible and can be implemented with multiple datasets to monitor ecosystems.

## Graphical abstract

Understanding complex datasets efficiently and easily using visualization tools is useful for quick decision‐making. The proposed interactive tool is designed to study the data structure, summary, correlations, and sampling efficiency. It also helps to understand the ecosystem structure, species richness patterns, and species–environment relationships. The R‐codes used in this tool are reproducible for datasets of multiple ecosystems. The tool could help planning for conservation be more meaningful and easy.

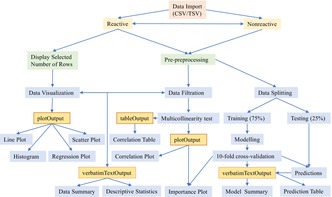

## 1 Introduction

Understanding the complexities in biodiversity and its monitoring is one of the fundamental goals of ecology. Mountains have high levels of environmental heterogeneity and offer a multitude of microhabitat conditions for the coexistence of more species. A relatively stable climate of a mountain acts as refugia for many endemic species (Nogués‐Bravo et al. 2007), and a larger geophysical extent offers more complexities. Irregular decreases in atmospheric pressure and soil temperature in mountain systems cause trade‐offs between air and soil. Apart from these trade‐offs between soil and air, the influences of terrain complexities on solar radiation, potential evaporation, temperature, and rainfall also significantly contribute to the intricacies in biodiversity of a mountain ecosystem. Each mountain is somehow unique and its climatic, biological, physiographic, and socioeconomic amplitudes are of special significance to its ecosystem maintenance and sustainability. Recently, the increases in human interference due to the amendments of land use patterns, the establishment of hydroelectric plants, and the increase in the agriculture and livestock population have created tremendous pressure on the biodiversity of the mountain.

A big mountain range like the Himalayas poses greater intricacies, a good number of microhabitat conditions, and endemism (Zachos and Habel 2011; Chitale and Behera 2019). This global biodiversity hotspot, with a plant distribution of up to 5500 m, is home to the world's highest peaks extending over 2400 km. Its climate is a trade‐off between tropical and temperate features, characterized by dry and short winters and less hot summers with little variation in the day (Mani 1974; Zobel and Singh 1997). Its overall tropical climate is driven by its low latitudinal position. The steep slopes with flat surfaces influence its species' existence and survival, where dispersal, isolation, and speciation contribute toward a richer biodiversity. The present study that considers an example dataset derived from a national biodiversity assessment database includes floral data from the Indian parts of the Western Himalayas.

The western Himalayas receive lower monsoonal rainfall in summer and moderate rainfall by westerlies in winter. Its rain and temperature can be as low as 10 to 70 mm and −45°C. The diversity of its plants is dominated by herbs, followed by shrubs and trees. Dryness, seasonality of temperature and precipitation, and precipitation of the driest quarter are key factors for its complex species diversity patterns (Panda et al. 2017; Panda 2018). Land degradation, population growth in the southern foothills, overgrazing, increase in livestock population, slash and burn agriculture, and tree cutting for fuel and trade have significantly influenced its biodiversity at large (Chandrasekhar, Singh, and Roy 2003; Sharma, Raghubanshi, and Singh 2005; Kharkwal 2009; Sharma, Suyal, et al. 2009; Sharma, Chettri, et al. 2009; Rashid et al. 2015; Tewari, Verma, and von Gadow 2017). In addition, studies of its biodiversity and similar mountain ecosystems have never been easy. A tool that provides instantaneous investigations on species richness patterns and species–environment relationships along elevational gradients has never been available. To the best of our knowledge, the impacts of environmental heterogeneity on biodiversity of mountain ecosystems through an automated monitoring procedure are attempted for the first time. This visualization tool is interactive with tremendous flexibility and helpful to stakeholders, instructors, researchers, ecologists, planners, and managers to monitor biodiversity easily. It allows users to visualize data and other related information instantly. The tool also helps users to communicate effectively, that is, crucial for biodiversity maintenance and sustainability.

## 2 Methods

A floral data set from 739 sample locations for 23 vegetation types (VT) of a national database, the ‘Biodiversity Characterization at the Landscape Level’ (Roy et al. 2012), was classified into categories of herb, shrub, and tree life forms. Initially, 27 environmental variables were acquired and assigned a common coordinate system of the 44°N UTM zone of the WGS84 datum of 1 km^2^ spatial resolution (Table 1). To investigate the data structure, the visualization tool provides options to verify samples using line plots and scatterplots. It allows users to study gradient patterns along vast elevation ranges by sliding over the sliding bar. Navigating further, the tool allows users to view data summary and descriptive statistics. It also provides flexibility to test multicollinearity interactively for different VTs and richness patterns. As a preliminary investigation, the tool offers both linear regression and a generalized additive model (Hastie and Tibshirani 1990) to understand relationships between a predictor variable and species richness. We used the package “mgcv” to derive generalized additive modeling (GAM) output (Wood and Wood 2015). The second‐order polynomial equation of the linear model is displayed along with the 95% confidence intervals for each regression curve. Users can interactively opt for a predictor, elevational range, and species‐rich patterns to visualize their relationships on the dashboard. Users can study impacts of multiple predictors on the richness of plants and their life forms using modeling protocols such as linear, generalized additive model, and random forest model (Breiman 2001). The results derived to understand these species–environment relationships are evaluated by splitting the original data into 75:25 training and testing datasets, followed by 10‐fold cross‐validation. Validation is done by using the testing dataset, and predictions of these models are compared and displayed. This web‐based visualization tool is constructed with the R Shiny package (Doi et al. 2016; Chen et al. 2018; Jia et al. 2022; Lammens et al. 2022; Chang et al. 2024) and the analyses are performed solely using R (R Core Team 2023).

**TABLE 1:** Description of physiographic, climate, soil, and disturbance variables.

Variable	Min	Max	Mean	SD
Physiography				
Elevation (m)	467	5468	2726.75	1178.56
Aspect (°)	0.18	359.75	192.96	111.64
Slope (°)	0.45	38.84	12.26	6.88
Terrain ruggedness index	66.52	1693.19	726.06	275.75
Climate				
Aridity Index	0.05	0.23	0.12	0.05
Mean diurnal temperature range (°C)	7.20	12	9.61	1.00
Isothermality (°C)	29	44	35.13	2.99
Mean annual temperature (°C)	−6.9	22.5	10.01	6.96
Mean annual precipitation (mm)	304	2796	1215.12	599.63
Precipitation of coldest quarter (mm)	60	407	188.69	68.88
Precipitation of driest quarter (mm)	39	190	100.91	32.79
Precipitation of driest month (mm)	6	31	15.54	6.14
Potential evapotranspiration (ha/year)	431	1526	953.05	223.31
Precipitation seasonality (CV)	37	132	69.69	30.31
Precipitation of wettest month (mm)	39	889	284.58	205.39
Precipitation of wettest quarter (mm)	81	1027	433.70	280.30
Precipitation of warmest quarter (mm)	81	1027	433.70	280.30
Temperature annual range (°C)	21.8	33.6	27.11	3.37
Temperature of coldest month (°C)	−23.8	7.3	−4.19	8.13
Temperature of coldest quarter (°C)	−17.1	14.4	1.77	7.92
Temperature of driest quarter (°C)	−10.5	18.9	6.44	6.93
Temperature seasonality (CV)	445.9	791.8	595.58	106.54
Temperature of wettest quarter (°C)	−9.1	27.2	12.02	9.67
Temperature of wettest month (°C)	9.70	37.30	22.92	6.09
Temperature of warmest quarter (°C)	3.10	29.70	16.97	5.76
Disturbance				
Global human footprint	10.00	73.00	30.61	11.10
Human appropriation of net primary productivity	2.10	130.51	22.01	16.89

**FIGURE 1:**
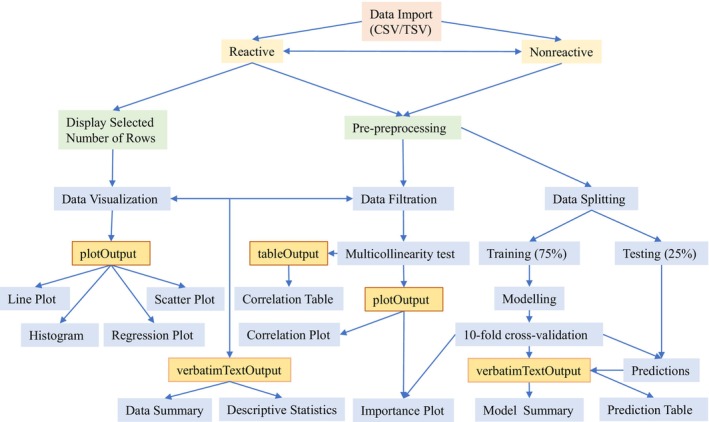
Flowchart shows the steps for constructing the visualization tool, where “plotOutput,” “tableOutput,” and “verbatimTextOutput” render plots, tables, and texts, respectively.

Primarily, the tool has two major components: a display division, that is, user interface (UI), and a functional component, that is, server. The display division takes care of dashboard design and skeleton of the whole framework. The user interface accommodates all instructions for the features discernable externally, where users can navigate, select, and see useful information. The server carries out data analyses, modeling, and output generation at the back‐end. The most of the functional keys of this tool are automated for users to investigate data structure and prepare outputs in the form of graphs, plots, and tables interactively. It displays an interactive view of plant species and life forms patterns of different VTs across elevational gradients. The tool generates data summary, descriptive statistics, and display correlations between predictor variables interactively (Panda 2023). We used the packages “psych” to derive descriptive statistics (Revelle 2024), “ggplot2,” “GGally” and “ggpubr” for plotting and graphs (Wickham 2016; Schloerke et al. 2024; Kassambara 2023), and “dplyr,” “dismo” and “caret” packages for data splitting, train data, set training control, and cross‐validation (Liaw and Wiener 2002; Hijmans et al. 2011; Kuhn 2008). The skeletal structure of the workflow is presented and the key steps used in construction of this web application are highlighted (Figure 1).

## 3 Results

### 3.1 Data Input and Visualization

**FIGURE 2:**
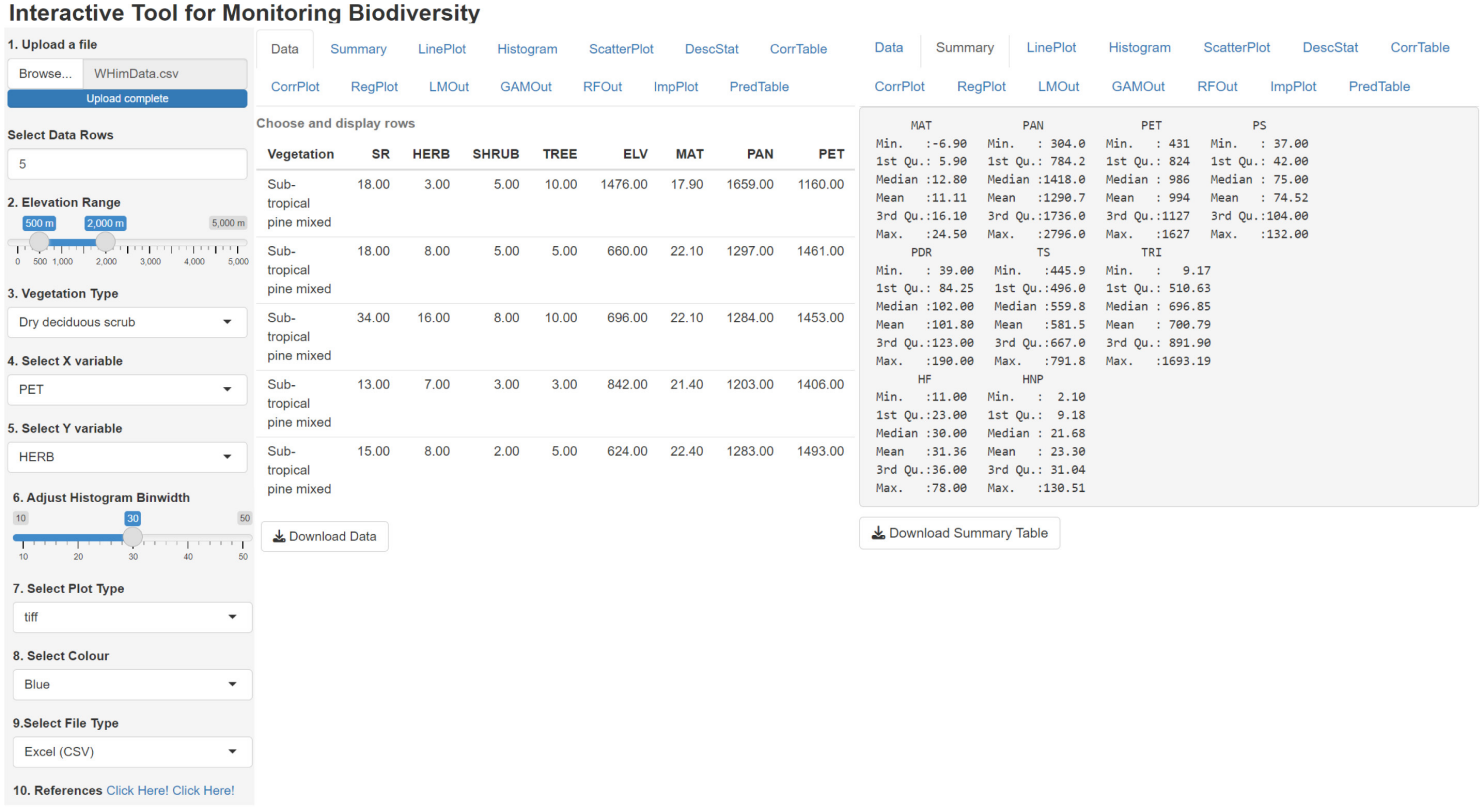
Framework depicts user's options at the left and top panels. On the left of the main panel, the table displays information about five rows of data for different vegetation types; and the right of the main panel highlights the data summary. SR, species richness, ELV, elevation; MAT, mean annual temperature; PAN, mean precipitation; PET, potential evapotranspiration; PS, precipitation seasonality; PDR, precipitation of the driest quarter; TS, temperature seasonality; TRI, terrain ruggedness index; HF, global human footprint; HNP, human appropriation of net primary productivity.

Data visualization involved dynamic import in txt/csv formats. The imported file displays the data structure, similar to the “view” or “head” functions in R. However, this visualization tool allows users to select a number of rows for displaying (Figure 2).

### 3.2 Data Structure and Filtering

**FIGURE 3:**
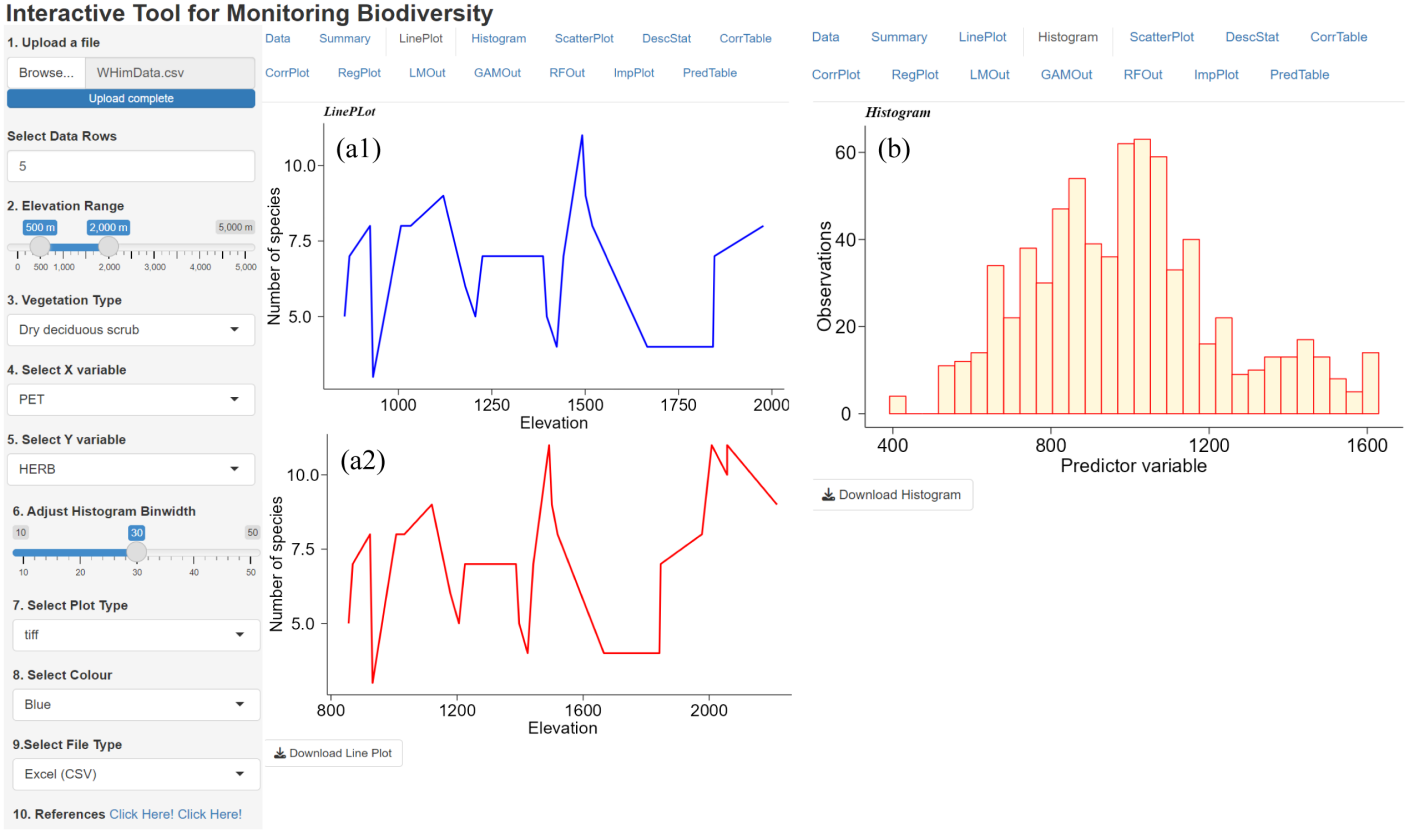
Frameworks depict (a1) line plots of herb richness patterns between 500 and 2000 m elevational ranges and (a2) between 800 and 2000 m elevational ranges; (b) represents a sample histogram plot of a predictor variable and its sample frequency.

**FIGURE 4:**
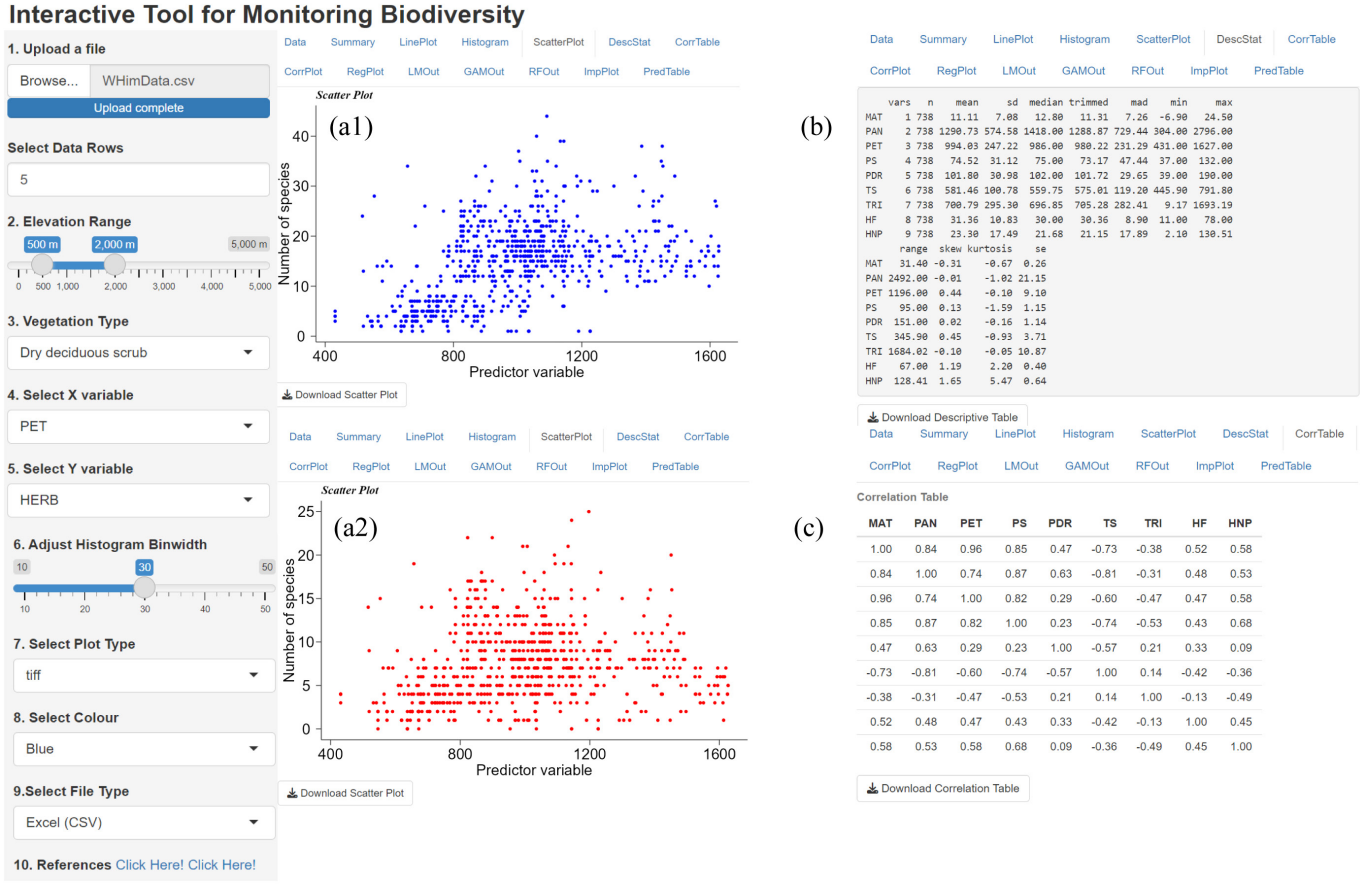
(a1 and a2) Scatterplots showing sample distribution points; (b) descriptive table explaining statistical information about the predictor variables; and (c) correlation table representing correlations between selected number of predictor variables. Refer to Figure 2 for abbreviations.

**FIGURE 5:**
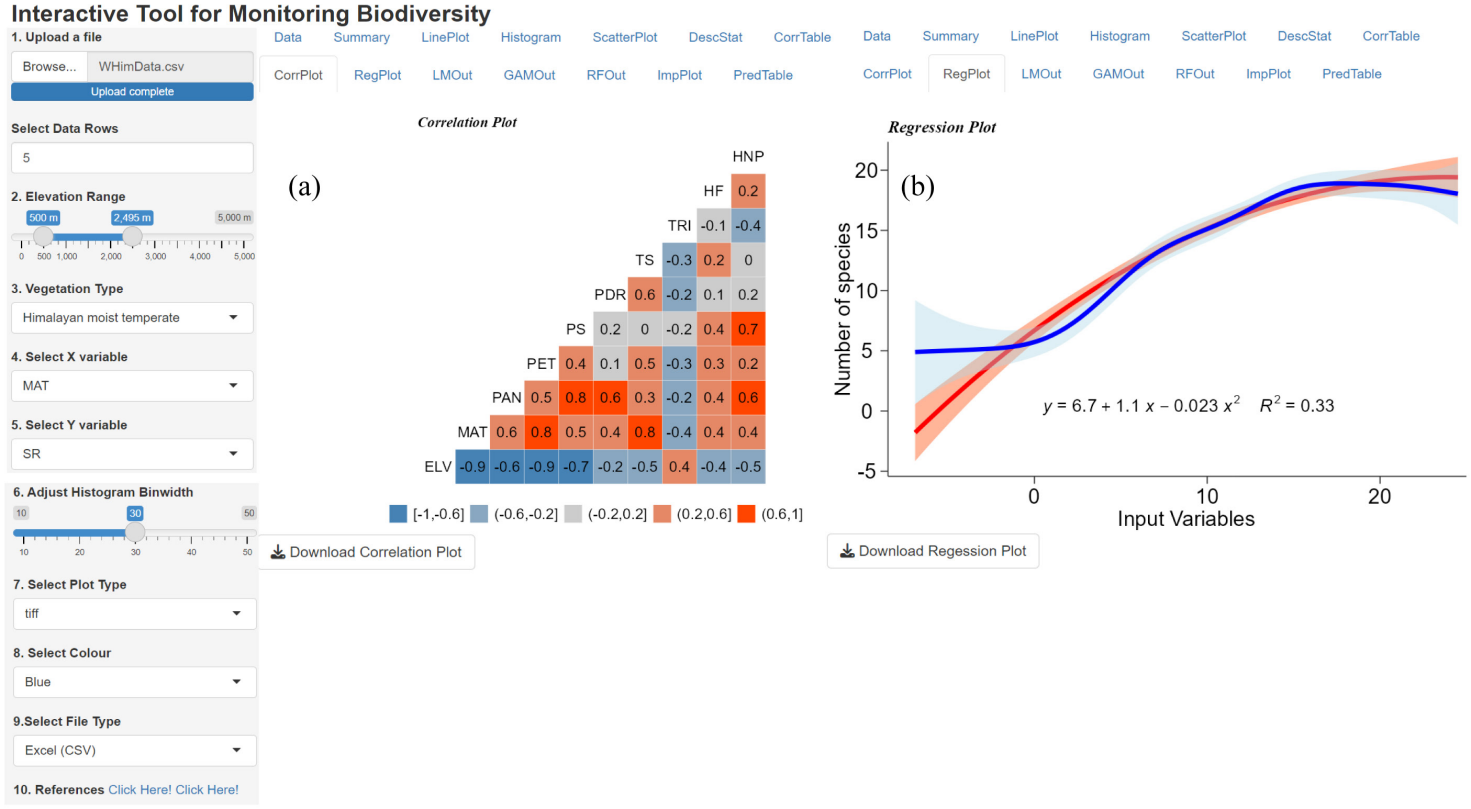
(a) Sample correlation plot; (b) Sample regression plot between potential evapotranspiration (PET) and herb richness compared using linear regression model and generalized additive model, where the regression curves are shown at 95% confidence intervals with a second‐order linear regression equation.

The original data are examined by generating line plots, histograms, and scatter diagrams (Figures 3 and 4). The correlation between predictor variables is generated interactively to test multicollinearity. To visualize the significance of each VT on plant or life form richness along an elevation gradient, the user dynamically opts for the elevational range, select the richness type, and predictor variable of interest to obtain regression curves by using linear and non‐linear models (Figure 5a).

### 3.3 Navigation

Navigation allows users to make choices, for example, VTs, plants or life form richness, an elevational range, predictor variables, and modeling. A slider is used to select a range to visualize the impacts of elevation on species richness patterns. The visualization tool helps to see richness patterns of VTs and understand their complexities along with multiple elevational ranges, which was never easy in traditional setups. Understanding richness patterns of VTs in multiple ranges explains intricacies in ecosystem functioning and assists conservationists and planners to monitor biodiversity easily and efficiently. Line plots, which display sampling distributions of predictor variables, can easily be navigated using the drop‐down menu. Similarly, scatter plots that represent the distribution of the variables of plant or life form richness can also be generated interactively and displayed.

### 3.4 Modeling and Predictions

**FIGURE 6:**
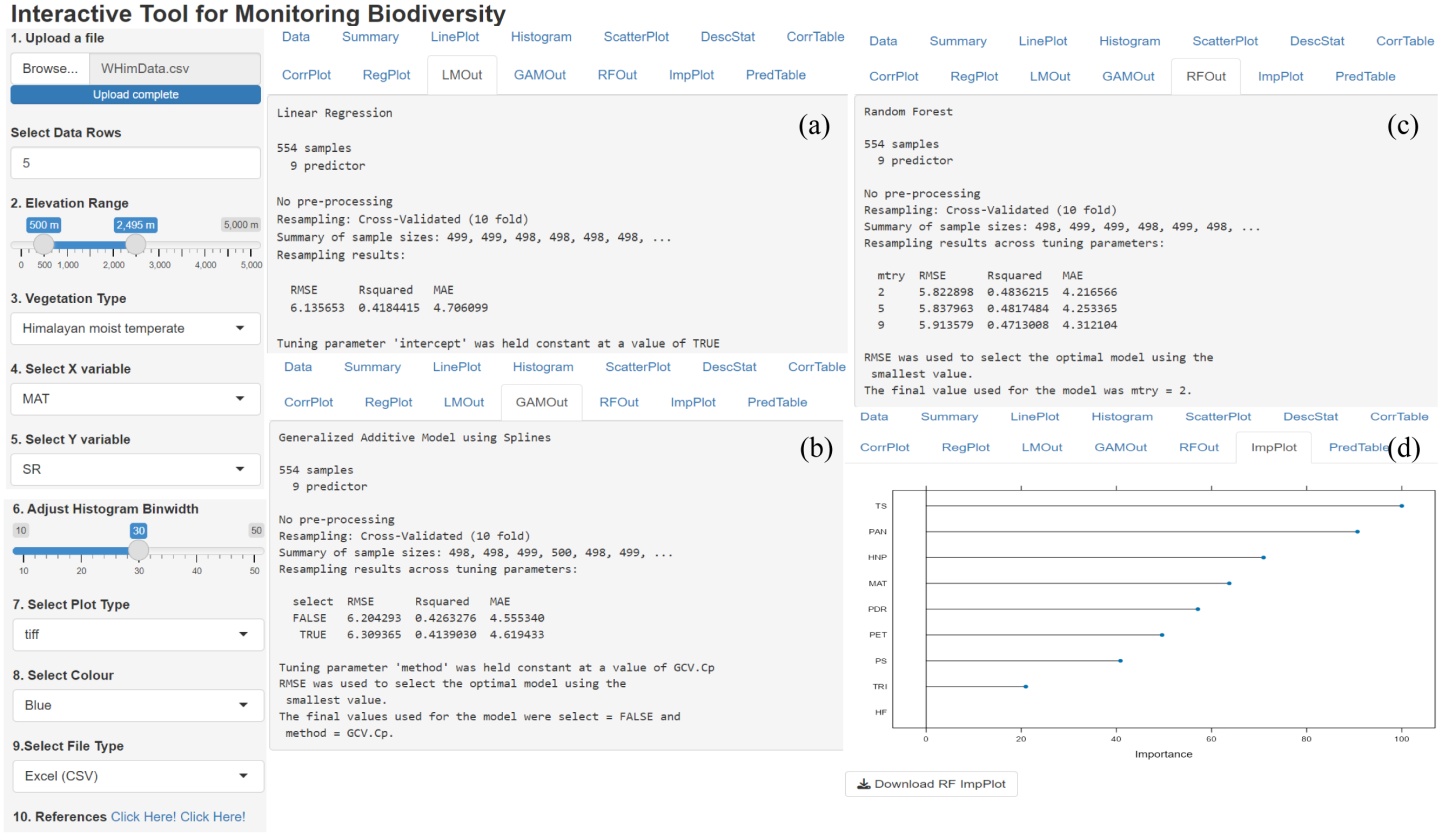
Cross‐validated outputs show species–environment relationships derived by a (a) linear regression model, (b) generalized additive model, (c) random forest model, and (d) the relative importance of variables in describing herb richness by the best‐performing random forest model.

**FIGURE 7:**
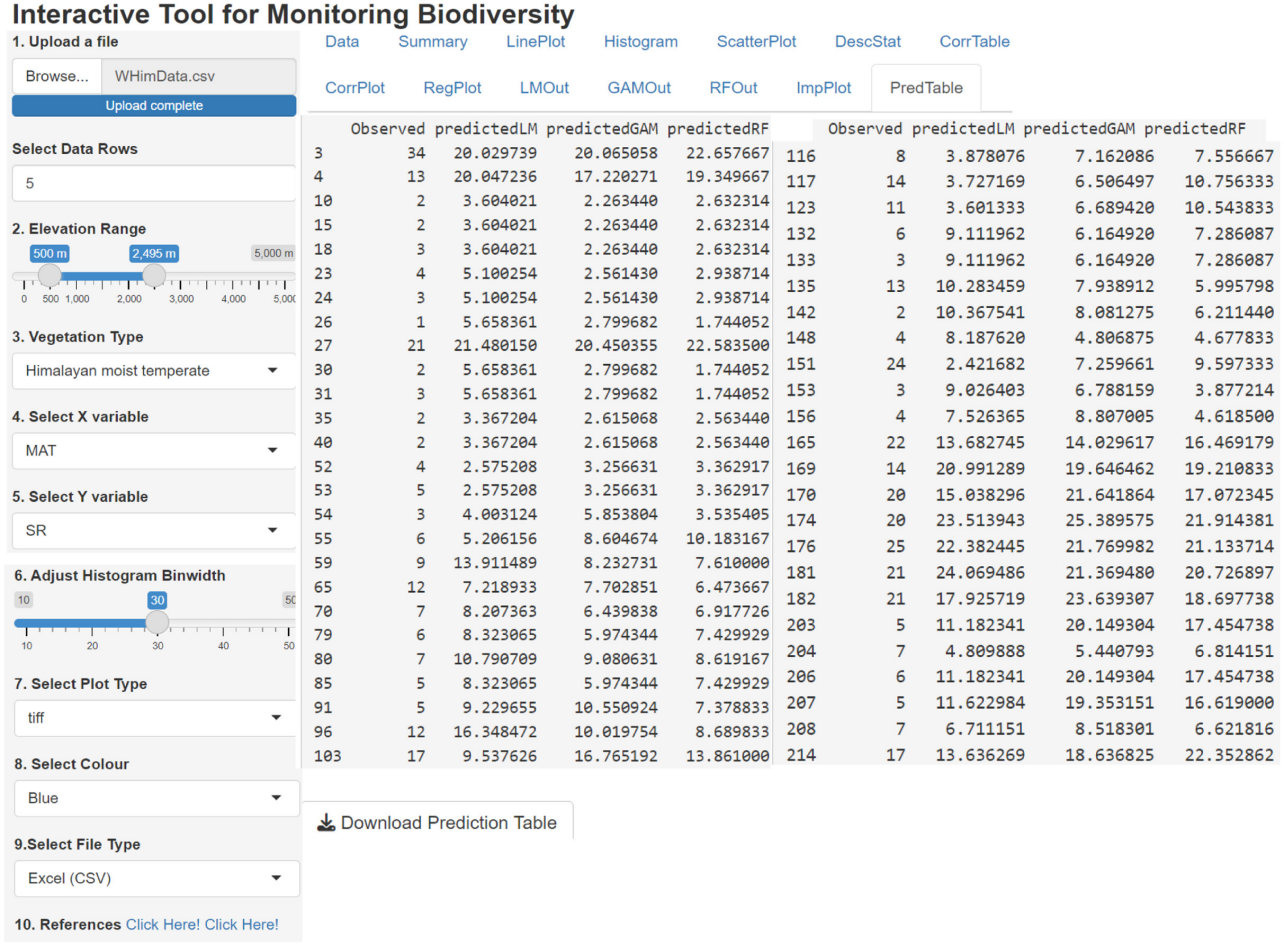
Original species richness data and the corresponding predicted values derived by the linear regression model, general additive model and random forest model.

Species–environment relationships of plant or life form richness for a single‐factor are compared by using simple linear regression and generalized additive model (Figure 5b). After initial data splitting, training/test datasets (75:25), trained models are evaluated by 10‐fold cross‐validation. The outputs of linear regression model, generalized additive model and random forest model are validated with the test dataset. The predicted results are tabulated and the random forest model derived importance plot showing significance of predictor variables in herb richness is displayed (Figure 6). Predicted outputs of three models are tabulated and displayed (Figure 7).

### 3.5 Format Options

The tool allows users to make choices for deriving table and text outputs in different file formats such as “txt” “excel (CSV),” and “doc.” The excel (CSV) format is set as the default option. Similarly, plot outputs can also be prepared in “png,” “jpeg” and “tif” formats. The png format is set as the default plotting option. The drop‐down menu helps users select the formats of their choice.

### 3.6 Color Options

This tool provides options for the users to choose colors for plot outputs: line plots, scatter plots, and histograms. Additionally, histograms have the flexibility to change border colors and bin size. The current version includes the colors “blue,” “green,” and “red,” where “blue” is set as the default color.

### References

3.7

The reference section in the tool provides options for users to see relevant literature by clicking on the given links.

## 4 Discussions

The web‐based application developed using open‐source R‐Shiny package displays outputs in the main panel and provides flexibility to navigate, select colors and file/plot formats. When objects are reactive, clicking on the select button, users can make choices for outputs. To see outputs, users need to click on the tabs of top panel. The tabs of the top panel are highlighted in the following order:

*Data* displays first five rows by default, but the flexibility to see the “n” number of rows is possible based on user's choice.

*Summary* is a table that highlights the minimum, mean, and median of each variable with 1st and 3rd interquartile ranges.

*LinePlot* dynamically visualizes species richness patterns of plants or life forms along an elevational range of a VT. Herb is set as the default life form category and the “dry deciduous scrub” as default VT and the “potential evapotranspiration (PET)” as a default predictor.

*Histogram* highlights observations of a predictor variable in the *y*‐axis and its corresponding observation values in the *x*‐axis. Users have options to select VT type, change fill colors, border colors, and bin length of histograms.

*ScatterPlot* dynamically displays the distribution of predictor variable for plant or life form richness in multiple plot formats and colors.

*DescStat* displays simple statistical details about data that includes standard deviation, standard error, skewness, and kurtosis.

*CorrTable* displays the correlation between predictor variables in tabular form. Users can visualize the correlation table of each VT and along an elevational range.

*CorrPlot* represents correlations between predictor variables of a selected group of a VT in plot format.

*RegPlot* describes the effect of each predictor variable on richness of plants or life forms. Users have options to compare outputs of both linear regression and GAM.

*LMOut* displays the cross‐validated outputs of multivariate linear regression model.

*GAMOut* demonstrates the cross‐validated outputs of GAM model.

*RFOut* shows the random forest model outputs after cross‐validation.

*ImpPlot* that shows importance among predictors in explaining plant richness is derived from random forest model.

*PredTable* A table that displays predictions after of validation. For the users' convenience, the predicted outputs of linear regression, GAM and random forest models are put together.

This interactive visualization tool for biodiversity monitoring is constructed solely using open‐source R. This tool allows users to investigate multiple data analyses, and species distribution patterns along elevation gradient. Species–environment relationships is made simple and understandable to users. The customization of outputs dynamically at the click of the button and to slide over elevational ranges quickly are special features of this application. This dynamic visualization of species richness patterns along an elevational gradient has never been easy by traditional methods. The tool helps users by displaying results on screen and with options to navigate easily. The flexibility to download outputs in different formats using color patterns from the drop‐down menu makes its use more meaningful. More interestingly, this visualization tool need not require special computational background for users to use it. On the other hand, it is essentially advancing our basic understanding of a mountain ecosystem and ecosystems of similar nature. The present tool provides a complete set of instructions for a general view of data, simple analysis, and modeling. A slight tweak in the present application could help in monitoring ecosystems for efficient planning and program implementation. The codes are available in the GitHub repositories and a full‐fledged R‐package is under development.

## 5 Conservation Implications

The biodiversity monitoring tool has tremendous flexibility for users. It helps in visualizing multiple datasets interactively and instantly. The tool has flexibilities to customize and can accommodate datasets from multiple sources. It has immense implications in conservation and monitoring biodiversity of multiple ecosystems. The R‐codes used in this tool preparation are editable and makes it easy to fit different datasets. Stakeholders, instructors, researchers, ecologists, planners, and managers can use it for free in first‐hand learning, data interpretation, and decision making. Biodiversity monitoring becomes easy with availability of multiple options for conservation planning. Data structure and methodology, essential for an accurate prediction, could be visualized instantly for quick and precise decision‐making. This tool improves the conservation strategy and policy implementation, and helps users to communicate effectively, crucial for biodiversity maintenance and sustainability.

## Author Contributions

**Rajendra Mohan Panda:** conceptualization (lead), data curation (lead), formal analysis (lead), investigation (lead), methodology (lead), project administration (lead), resources (lead), software (lead), validation (lead), visualization (lead), writing – original draft (lead), writing – review and editing (equal). **Padmanava Dash:** funding acquisition (lead), project administration (lead), resources (supporting), supervision (supporting), writing – review and editing (supporting). **Partha Sarathi Roy:** resources (supporting), supervision (equal), writing – review and editing (supporting).

## Conflicts of Interest

The authors declare no conflicts of interest.

## Supporting information


Data S1.

## Data Availability

Data and R‐codes are available.
