# Supplementary material for: A Novel Web‐Based Approach for Monitoring Biodiversity
Source: Ecol Evol. 2024 Sep 29;14(10):e70364. doi: 10.1002/ece3.70364 (PMC11439740; doi:10.1002/ece3.70364)
Supplement: Supplementary file 1 — Data S1. [file ECE3-14-e70364-s001.zip › WHimData.pdf]

Vegetation	SR	HERB	SHRUB	TREE	ELV	MAT	PAN	PET	PS	PDR	TS
Sub-tropical pine mixed	18	3	5	10	1476	17.9	1659	1160	98	117	536.6
Sub-tropical pine mixed	18	8	5	5	660	22.1	1297	1461	110	86	614.7
Sub-tropical pine mixed	34	16	8	10	696	22.1	1284	1453	110	85	613.9
Sub-tropical pine mixed	13	7	3	3	842	21.4	1203	1406	110	83	599.5
Sub-tropical pine mixed	15	8	2	5	624	22.4	1283	1493	112	85	625.4
Pine	19	11	1	7	647	22.2	1258	1477	113	82	621
Dry alpine pasture	3	3	0	0	4392	-0.2	334	682	38	42	776.5
Dry alpine pasture	3	2	1	0	4392	-0.2	334	682	38	42	776.5
Dry alpine pasture	5	3	1	1	4392	-0.2	334	682	38	42	776.5
Dry alpine pasture	2	2	0	0	4430	-0.3	337	672	37	43	774.8
Dry alpine pasture	2	1	1	0	4430	-0.3	337	672	37	43	774.8
Dry alpine pasture	2	1	1	0	4430	-0.3	337	672	37	43	774.8
Dry alpine pasture	4	1	1	2	4430	-0.3	337	672	37	43	774.8
Dry alpine pasture	3	2	0	1	4430	-0.3	337	672	37	43	774.8
Dry alpine pasture	2	1	1	0	4430	-0.3	337	672	37	43	774.8
Pine	13	7	1	5	682	22.3	1288	1478	109	87	617.4
Dry alpine pasture	3	2	1	0	4430	-0.3	337	672	37	43	774.8
Dry alpine pasture	3	2	0	1	4430	-0.3	337	672	37	43	774.8
Dry alpine pasture	1	0	1	0	4430	-0.3	337	672	37	43	774.8
Dry alpine pasture	3	2	1	0	4437	-1	331	652	37	41	777
Dry alpine pasture	3	2	0	1	4437	-1	331	652	37	41	777
Dry alpine pasture	3	2	0	1	4437	-1	331	652	37	41	777
Dry alpine pasture	4	3	1	0	4437	-1	331	652	37	41	777
Dry alpine pasture	3	2	1	0	4437	-1	331	652	37	41	777
Dry alpine pasture	1	0	1	0	4566	-0.6	341	637	37	43	777.1
Dry alpine pasture	1	1	0	0	4566	-0.6	341	637	37	43	777.1
Sub-tropical pine mixed	21	11	2	7	1000	20.7	1533	1340	118	92	581.2
Dry alpine pasture	2	1	1	0	4566	-0.6	341	637	37	43	777.1
Dry alpine pasture	2	0	1	1	4566	-0.6	341	637	37	43	777.1
Dry alpine pasture	2	0	1	1	4566	-0.6	341	637	37	43	777.1
Dry alpine pasture	3	2	1	0	4566	-0.6	341	637	37	43	777.1
Dry alpine pasture	4	1	1	1	4521	-1	339	646	37	43	779.8
Dry alpine pasture	2	2	0	0	4521	-1	339	646	37	43	779.8
Dry alpine pasture	2	2	0	0	4521	-1	339	646	37	43	779.8
Dry alpine pasture	2	1	0	0	4981	-3.7	322	546	42	41	781.2
Dry alpine pasture	3	2	1	0	4981	-3.7	322	546	42	41	781.2
Dry alpine pasture	2	0	2	0	4981	-3.7	322	546	42	41	781.2
Sub-tropical pine mixed	18	9	2	7	827	21.4	1397	1401	117	85	595.4
Dry alpine pasture	2	2	0	0	4981	-3.7	322	546	42	41	781.2
Dry alpine pasture	2	2	0	0	4981	-3.7	322	546	42	41	781.2
Dry alpine pasture	3	3	0	0	4859	-2.9	335	565	41	42	779
Dry alpine pasture	4	4	0	0	4859	-2.9	335	565	41	42	779
Dry alpine pasture	2	2	0	0	4795	-2.4	342	574	40	42	778.9
Dry alpine pasture	2	2	0	0	4795	-2.4	342	574	40	42	778.9
Dry alpine pasture	2	2	0	0	4795	-2.4	342	574	40	42	778.9
Dry alpine pasture	2	1	0	1	4795	-2.4	342	574	40	42	778.9

Dry alpine pasture	2	2	0	0	4795	-2.4	342	574	40	42	778.9
Dry alpine pasture	2	1	0	1	4581	-1.6	352	619	37	45	773.8
Scrub	23	9	1	13	728	21.9	1443	1441	118	85	609.9
Dry alpine pasture	3	3	0	0	5468	-6.9	304	431	48	39	791.8
Dry alpine pasture	4	4	0	0	5468	-6.9	304	431	48	39	791.8
Dry alpine pasture	4	4	0	0	5468	-6.9	304	431	48	39	791.8
Dry alpine pasture	5	4	0	1	5468	-6.9	304	431	48	39	791.8
Dry alpine pasture	3	3	0	0	4820	-3.1	379	541	40	46	772.4
Dry alpine pasture	6	6	0	0	4472	-0.5	452	634	39	53	766.2
Dry alpine scrub	11	8	0	3	2728	9.4	1047	921	42	120	659.5
Dry alpine scrub	13	5	4	4	2728	9.4	1047	921	42	120	659.5
Dry alpine scrub	4	2	1	0	4313	0.1	573	591	41	64	741
Dry alpine scrub	9	4	3	1	2717	9.3	1022	919	42	118	661.8
Sub-tropical pine mixed	26	9	3	13	798	21.8	1478	1422	118	85	605.2
Dry alpine scrub	5	1	3	0	2717	9.3	1022	919	42	118	661.8
Dry alpine pasture	14	7	3	2	3268	6.6	787	846	46	89	694.9
Dry alpine pasture	21	16	2	3	3268	6.6	787	846	46	89	694.9
Dry alpine pasture	15	4	5	5	3520	5.8	725	799	46	82	702.7
Dry alpine pasture	12	3	5	3	3788	3.7	616	760	43	70	723.2
Dry alpine pasture	8	4	3	0	3226	6.8	788	854	48	91	685.1
Dry alpine pasture	12	9	3	0	3537	5.1	689	803	46	77	706.5
Dry alpine pasture	7	5	1	1	3917	3.4	558	761	42	66	722.5
Dry alpine pasture	11	8	2	0	3917	3.4	558	761	42	66	722.5
Dry alpine pasture	7	6	0	1	4014	2.6	632	724	39	74	700.5
Pine	26	14	5	7	1944	16.2	1563	1041	96	114	524
Tropical Moist deciduous	16	8	4	4	850	21.1	1766	1370	122	96	588
Dry alpine pasture	6	4	0	2	4014	2.6	632	724	39	74	700.5
Dry alpine pasture	6	5	1	0	4014	2.6	632	724	39	74	700.5
Dry alpine pasture	8	6	1	1	4014	2.6	632	724	39	74	700.5
Dry alpine pasture	6	4	1	1	4014	2.6	632	724	39	74	700.5
Dry alpine pasture	7	6	0	1	4014	2.6	632	724	39	74	700.5
Dry alpine pasture	10	9	0	1	4014	2.6	632	724	39	74	700.5
Dry alpine pasture	6	3	1	2	3883	2.9	640	749	39	74	695.7
Dry alpine pasture	7	3	2	2	3621	5.4	747	801	42	86	673.5
Dry alpine pasture	5	2	1	2	3883	2.9	640	749	39	74	695.7
Dry alpine pasture	10	7	1	2	3883	2.9	640	749	39	74	695.7
Tropical Moist deciduous	26	10	5	11	812	21.3	1712	1379	121	95	594.7
Dry alpine pasture	8	6	1	1	3883	2.9	640	749	39	74	695.7
Dry alpine pasture	5	3	1	1	3883	2.9	640	749	39	74	695.7
Dry alpine pasture	4	3	0	1	3385	6.4	801	839	44	93	662.4
Dry alpine pasture	8	5	0	3	3621	5.4	747	801	42	86	673.5
Dry alpine pasture	7	5	0	2	3385	6.4	801	839	44	93	662.4
Dry alpine pasture	8	7	1	0	3385	6.4	801	839	44	93	662.4
Dry alpine pasture	12	10	1	0	3385	6.4	801	839	44	93	662.4
Dry alpine pasture	5	5	0	0	3676	3.4	765	718	41	83	686.4
Dry alpine pasture	5	4	1	0	3539	4.1	796	732	40	86	680.9
Temperate coniferous	5	4	0	1	2603	11	1275	950	43	137	649

Pine	15	7	1	7	834	21.5	1804	1386	122	97	596.7
Dry alpine scrub	7	1	1	5	2807	9.2	1048	925	41	117	676.9
Temperate coniferous	12	0	8	1	2605	11	1276	954	43	137	649.8
Dry alpine scrub	15	7	6	2	4682	-1.4	437	592	39	52	765.4
Dry alpine pasture	8	1	3	4	3733	4.4	657	765	43	74	725.5
Dry alpine pasture	12	5	1	5	3062	8.5	905	892	44	104	694.1
Dry alpine pasture	6	6	0	0	3062	8.5	905	892	44	104	694.1
Dry alpine pasture	6	5	0	1	3062	8.5	905	892	44	104	694.1
Dry alpine scrub	8	7	0	1	3495	5.3	687	807	45	78	728.8
Dry alpine pasture	17	11	6	0	3190	5.9	718	834	45	81	723.2
Dry alpine pasture	17	11	4	2	3624	5	686	769	44	77	726.9
Pine	28	10	3	15	606	22.4	1963	1450	122	103	619.8
Dry alpine pasture	25	16	3	6	3624	5	686	769	44	77	726.9
Dry alpine pasture	21	10	8	3	3175	6.2	776	848	44	87	701.6
Dry alpine pasture	12	7	2	3	3175	6.2	776	848	44	87	701.6
Dry alpine scrub	10	5	0	5	2738	10.6	1226	929	42	135	643.3
Dry alpine scrub	15	10	5	0	2738	10.6	1226	929	42	135	643.3
Dry alpine pasture	19	6	6	7	3286	5.8	748	811	44	84	707.3
Dry alpine scrub	24	14	1	9	3202	6.7	830	832	43	94	693.9
Dry alpine pasture	14	6	6	2	4174	1.3	522	685	39	61	737.9
Dry alpine pasture	10	8	2	0	4410	-0.4	451	649	38	54	757.9
Tropical Moist deciduous	11	5	0	6	779	21.6	1697	1396	122	92	600.5
Dry alpine pasture	8	4	2	2	4468	-1.5	444	627	39	54	748.5
Dry alpine pasture	14	7	3	4	4767	-2.4	426	562	40	51	752.6
Dry alpine pasture	3	3	0	0	3189	7	785	865	50	91	679.4
Dry alpine pasture	4	4	0	0	3189	7	785	865	50	91	679.4
Dry alpine pasture	4	3	0	1	3189	7	785	865	50	91	679.4
Dry alpine pasture	4	3	0	1	3189	7	785	865	50	91	679.4
Dry alpine pasture	11	5	4	2	3189	7	785	865	50	91	679.4
Dry alpine pasture	11	7	4	0	4802	-2.3	435	578	40	52	748.8
Dry alpine pasture	8	5	3	0	4340	-0.7	469	640	38	56	741.2
Tropical Moist deciduous	15	9	1	4	633	22.2	1503	1465	121	85	618.2
Dry alpine pasture	6	5	0	1	3627	4.1	595	789	44	70	716.3
Dry alpine pasture	9	4	4	1	4440	-0.2	454	640	39	55	748
Dry alpine pasture	5	1	1	3	4575	-1	447	619	38	54	749.4
Dry alpine pasture	5	5	0	0	3095	7.6	824	884	50	96	673.4
Dry alpine pasture	5	5	0	0	3303	5.7	706	829	48	80	694.7
Dry alpine pasture	5	4	0	1	3095	7.6	824	884	50	96	673.4
Dry alpine pasture	6	4	1	1	3303	5.7	706	829	48	80	694.7
Dry alpine pasture	3	3	0	0	3303	5.7	706	829	48	80	694.7
Dry alpine pasture	4	3	1	0	2987	8.2	868	890	49	103	667.3
Dry alpine pasture	13	9	3	1	3252	7.1	808	860	48	93	677.5
Tropical Moist deciduous	22	9	4	8	619	22.3	1514	1472	120	85	623.2
Dry alpine pasture	4	3	0	1	4935	-3.8	321	532	39	41	791.2
Dry alpine scrub	2	2	0	0	3007	8.4	909	895	47	108	657
Dry alpine pasture	3	3	0	0	3947	2.3	593	722	40	68	714
Dry alpine scrub	2	2	0	0	4223	1.9	572	699	40	66	719.9

Dry alpine pasture	3	3	0	0	3947	2.3	593	722	40	68	714
Dry alpine pasture	2	2	0	0	3212	7.1	824	858	47	95	670.1
Dry alpine pasture	3	3	0	0	3111	7.7	858	875	47	100	663.2
Dry alpine pasture	4	4	0	0	3111	7.7	858	875	47	100	663.2
Dry alpine pasture	5	3	2	0	3666	4.1	658	768	43	75	698.5
Dry alpine pasture	7	5	1	1	3322	6.2	763	836	48	86	678.1
Sub-tropical pine mixed	38	20	7	11	646	22.1	1488	1451	118	86	614.4
Dry alpine pasture	4	4	0	0	3666	4.1	658	768	43	75	698.5
Dry alpine pasture	3	3	0	0	3666	4.1	658	768	43	75	698.5
Dry alpine pasture	5	5	0	0	4664	-1.2	439	617	41	53	743.4
Dry alpine pasture	24	14	6	4	5139	-4.2	422	516	47	50	751.8
Dry alpine pasture	1	1	0	0	4057	1.6	600	704	39	71	705.2
Dry alpine pasture	3	3	0	0	3764	4.5	721	781	39	86	678.8
Dry alpine pasture	3	1	1	1	3764	4.5	721	781	39	86	678.8
Dry alpine pasture	2	2	0	0	3764	4.5	721	781	39	86	678.8
Dry alpine pasture	4	4	0	0	4383	0.2	563	656	41	66	713.2
Dry alpine pasture	5	5	0	0	3903	2.8	673	736	39	78	688.6
Scrub	27	12	3	12	671	21.9	2095	1440	124	108	607.9
Dry alpine pasture	14	10	3	0	3676	4.5	741	781	40	86	672.5
Dry alpine pasture	7	6	0	1	3576	4.3	734	780	40	84	677.1
Dry alpine pasture	16	6	4	6	3772	4.6	711	782	41	82	678.4
Dry alpine scrub	6	5	1	0	3204	7.3	855	866	44	100	658.7
Dry alpine pasture	8	7	0	1	3712	3.8	683	771	40	80	689.1
Dry alpine pasture	3	2	0	1	3712	3.8	683	771	40	80	689.1
Temperate coniferous	22	14	5	3	3420	6.9	868	831	42	99	650.6
Pine	25	16	4	4	3029	9.9	1350	852	52	138	575
Temperate coniferous	23	16	2	5	2729	10.8	1460	931	54	147	576.8
Dry alpine pasture	14	9	2	3	4720	-0.8	510	606	41	59	728.2
Scrub	14	6	6	2	630	22.3	2158	1447	125	110	617.4
Temperate coniferous	20	14	1	4	3124	8.3	1116	864	41	124	603.4
Temperate coniferous	17	11	2	3	1632	16.1	1719	1120	69	159	557.6
Temperate coniferous	23	9	5	2	1632	16.1	1719	1120	69	159	557.6
Temperate coniferous	34	19	5	7	1632	16.1	1719	1120	69	159	557.6
Temperate coniferous	20	15	2	3	2069	13.9	1976	1031	79	170	560.6
Temperate coniferous	23	14	4	4	2467	11.6	1642	979	63	157	569.8
Temperate coniferous	25	17	3	5	2860	10.9	1491	834	54	150	582.1
Temperate coniferous	27	16	5	4	1567	16.8	1582	1143	63	152	559.4
Temperate coniferous	18	11	2	2	1567	16.8	1582	1143	63	152	559.4
Temperate coniferous	21	10	4	7	2299	13.7	1682	942	91	127	517.5
Himalayan moist temperate	11	7	2	2	1150	19.8	2476	1222	119	146	553.5
Temperate coniferous	21	12	3	4	1580	17	1571	1150	64	151	555.3
Dry alpine scrub	21	11	4	5	2076	13.7	1045	1056	43	121	545.8
Pine	20	11	4	5	1956	15.2	1158	1082	49	126	546.2
Temperate coniferous	23	12	2	6	2401	13	943	1056	38	118	543.8
Temperate coniferous	17	9	3	5	2401	13	943	1056	38	118	543.8
Dry alpine scrub	11	10	1	0	3481	5.9	929	823	41	122	602
Dry alpine scrub	18	14	1	3	3481	5.9	929	823	41	122	602

Dry alpine scrub	22	14	5	2	3481	5.9	929	823	41	122	602
Dry alpine scrub	27	22	4	1	3481	5.9	929	823	41	122	602
Dry alpine scrub	22	15	6	1	3481	5.9	929	823	41	122	602
Tropical Moist deciduous	21	7	3	11	1211	19.5	2396	1204	116	146	546.6
Temperate coniferous	23	19	2	2	1567	16.8	1582	1143	63	152	559.4
Temperate coniferous	13	9	3	1	1567	16.8	1582	1143	63	152	559.4
Dry alpine scrub	11	8	2	0	3583	5.4	932	810	42	120	603.4
Dry alpine scrub	17	12	3	2	3583	5.4	932	810	42	120	603.4
Dry alpine scrub	18	13	3	2	3583	5.4	932	810	42	120	603.4
Himalayan moist temperate	15	12	2	1	1791	16.1	1699	1063	88	138	535.6
Dry alpine scrub	18	15	2	1	3737	4.2	932	785	46	114	617.9
Dry alpine scrub	16	12	3	1	3737	4.2	932	785	46	114	617.9
Scrub	14	12	2	0	1688	17.1	1462	1119	77	124	541.2
Scrub	17	11	4	2	3374	5.9	962	786	41	103	628.3
Temperate coniferous	23	9	7	6	2424	13.2	1929	982	83	161	546.1
Dry alpine scrub	5	4	1	0	3919	3	907	752	50	107	627.8
Dry alpine pasture	7	5	1	1	4299	0.5	482	723	45	60	731.7
Dry alpine pasture	6	5	1	0	4694	-1.5	504	609	43	60	728
Dry alpine scrub	6	5	1	0	3919	3	907	752	50	107	627.8
Dry alpine scrub	5	4	1	0	3721	4.1	934	794	47	114	616.7
Dry alpine pasture	7	7	0	0	4384	0.4	627	694	50	75	694.5
Pine	21	17	2	2	3129	8	849	877	40	123	578.1
Dry alpine pasture	12	8	3	0	3129	8	849	877	40	123	578.1
Dry alpine scrub	24	12	3	8	3590	6.7	903	824	39	123	591.2
Dry alpine scrub	21	13	3	5	3590	6.7	903	824	39	123	591.2
Temperate coniferous	22	12	7	2	1690	16.1	2120	1074	97	160	529
Dry alpine pasture	17	10	4	2	3590	6.7	903	824	39	123	591.2
Dry alpine scrub	20	11	4	5	3590	6.7	903	824	39	123	591.2
Dry alpine scrub	25	17	4	4	3590	6.7	903	824	39	123	591.2
Dry alpine scrub	12	7	3	2	3592	6.5	908	822	39	123	593
Dry alpine scrub	11	4	3	4	3592	6.5	908	822	39	123	593
Dry alpine scrub	14	8	2	4	3551	5.9	929	822	41	122	599.3
Dry alpine scrub	15	7	4	4	3551	5.9	929	822	41	122	599.3
Dry alpine scrub	14	8	4	2	3551	5.9	929	822	41	122	599.3
Dry alpine scrub	18	14	2	2	3551	5.9	929	822	41	122	599.3
Dry alpine scrub	18	9	3	6	3551	5.9	929	822	41	122	599.3
Dry alpine pasture	14	7	4	1	4621	-2.1	436	579	39	52	763.6
Dry alpine scrub	12	6	4	2	3551	5.9	929	822	41	122	599.3
Pine	16	12	2	2	3180	7.7	856	871	39	123	579.2
Temperate coniferous	17	10	3	3	3180	7.7	856	871	39	123	579.2
Temperate coniferous	17	14	2	0	3761	4.8	968	786	44	121	608.7
Pine	13	10	2	1	3240	8	852	867	39	123	575.5
Temperate coniferous	12	10	1	1	3214	7.7	852	865	39	123	581.2
Dry alpine pasture	7	4	1	2	3214	7.7	852	865	39	123	581.2
Temperate coniferous	15	9	4	2	3129	8	849	877	40	123	578.1
Temperate coniferous	17	11	4	2	3214	7.7	852	865	39	123	581.2
Sub-tropical pine mixed	10	4	2	4	1501	17.8	1942	1145	94	147	541.8

Dry alpine pasture	13	9	2	2	3214	7.7	852	865	39	123	581.2
Dry alpine pasture	13	8	4	1	3214	7.7	852	865	39	123	581.2
Dry alpine pasture	16	7	5	4	3214	7.7	852	865	39	123	581.2
Temperate coniferous	24	17	4	3	3214	7.7	852	865	39	123	581.2
Temperate coniferous	25	15	6	4	3129	8	849	877	40	123	578.1
Temperate coniferous	25	13	7	5	3129	8	849	877	40	123	578.1
Temperate coniferous	22	13	6	3	3129	8	849	877	40	123	578.1
Pine	18	13	4	1	3129	8	849	877	40	123	578.1
Dry alpine pasture	16	12	3	1	2816	9.5	828	924	40	119	562.1
Dry alpine scrub	10	6	2	2	2784	9.7	824	930	40	119	564
Sub-tropical pine mixed	23	9	6	7	1809	15.5	1987	1066	97	154	531.5
Temperate coniferous	13	6	4	3	2374	12.2	785	1034	41	112	553.2
Temperate coniferous	19	12	2	5	2374	12.2	785	1034	41	112	553.2
Pine	15	11	2	2	3212	7.3	865	861	38	123	595.6
Temperate coniferous	19	12	5	2	2673	10.7	771	949	45	113	566.4
Temperate coniferous	15	13	1	1	2661	11.1	761	969	46	112	567
Temperate coniferous	8	3	2	3	2251	13.2	834	1037	39	113	556.7
Temperate coniferous	16	8	4	3	2497	12.5	714	1018	49	95	563.4
Dry alpine pasture	7	6	1	0	2362	11.4	721	1022	49	97	569
Dry alpine scrub	7	5	1	1	2358	11.9	719	1017	49	98	569.1
Sub-tropical pine mixed	17	4	4	8	1809	15.5	1987	1066	97	154	531.5
Dry alpine scrub	5	1	2	2	2480	12	710	1012	49	94	569.1
Dry alpine pasture	12	5	3	4	3103	8.9	755	924	45	113	598
Dry alpine scrub	23	14	4	5	2676	10.6	726	975	49	98	582.3
Dry alpine pasture	23	12	7	4	3450	5.7	851	844	39	114	629.1
Dry alpine pasture	19	12	4	3	3450	5.7	851	844	39	114	629.1
Dry alpine pasture	5	4	1	0	3129	8.2	784	893	42	115	602.9
Dry alpine scrub	17	13	3	0	3233	7.3	799	885	41	114	612.8
Dry alpine scrub	8	5	0	3	3080	8.5	775	913	44	114	600.7
Dry alpine pasture	1	1	0	0	2408	12.6	728	1034	47	106	563.1
Dry alpine pasture	1	1	0	0	2737	12.3	712	980	50	92	576.1
Temperate coniferous	18	7	5	5	2054	13.6	1974	1011	87	162	540.1
Dry alpine pasture	5	4	1	0	2539	11.2	698	1018	51	87	589.3
Dry alpine pasture	4	1	2	1	2579	10.5	689	1008	51	88	602.1
Dry alpine pasture	5	4	1	0	2639	11.2	689	1024	51	86	597.5
Dry alpine pasture	7	7	0	0	2820	9.4	671	980	50	92	624.5
Dry alpine pasture	1	1	0	0	3949	2.5	550	784	41	71	712.3
Dry alpine scrub	15	9	5	0	3443	6.1	713	875	40	95	665.9
Dry alpine pasture	21	15	5	1	3620	5.9	721	847	39	96	664.8
Dry alpine pasture	4	3	0	1	3561	5.6	750	844	39	98	664.3
Dry alpine pasture	6	4	0	0	3600	4.9	752	830	38	96	667.4
Temperate coniferous	16	8	3	5	2475	12.7	1847	964	80	158	549.3
Dry alpine pasture	6	6	0	0	3960	3.3	626	791	38	80	695.6
Dry alpine pasture	5	4	0	0	3878	3.7	644	807	39	82	689.6
Dry alpine pasture	5	4	0	1	3961	2.9	594	774	39	74	705.4
Dry alpine pasture	7	7	0	0	3975	2.4	586	753	39	73	710.2
Dry alpine pasture	10	7	2	0	4280	1.1	504	723	39	63	725.9

Dry alpine pasture	5	2	1	2	4322	0.7	497	704	40	61	730
Dry alpine pasture	3	3	0	0	4396	0.1	481	687	41	59	736.3
Dry alpine pasture	7	7	0	0	4396	0.1	481	687	41	59	736.3
Dry alpine pasture	14	8	1	3	4396	0.1	481	687	41	59	736.3
Himalayan moist temperate	21	7	4	9	1873	15.7	1509	1039	92	114	520.2
Temperate coniferous	19	10	4	4	2373	12.3	1783	954	76	156	553.3
Dry alpine pasture	4	4	0	0	4396	0.1	481	687	41	59	736.3
Dry alpine pasture	7	6	1	0	4408	-0.1	475	679	42	58	737
Dry alpine pasture	6	6	0	0	4438	-0.2	476	671	42	58	737.7
Dry alpine pasture	11	7	2	0	4467	-0.4	470	670	42	58	737
Dry alpine pasture	7	5	1	0	4438	-0.2	476	671	42	58	737.7
Dry alpine pasture	5	2	1	1	4467	-0.4	470	670	42	58	737
Dry alpine pasture	5	4	0	0	4467	-0.4	470	670	42	58	737
Dry alpine pasture	7	5	2	0	4515	-0.6	464	666	43	56	739.7
Dry alpine pasture	9	8	0	0	4396	0.1	481	687	41	59	736.3
Dry alpine pasture	5	4	0	1	4396	0.1	481	687	41	59	736.3
Temperate coniferous	15	6	5	4	2475	12.7	1847	964	80	158	549.3
Dry alpine pasture	4	4	0	0	4483	-0.2	490	677	43	60	731.9
Dry alpine pasture	4	3	0	1	4483	-0.2	490	677	43	60	731.9
Dry alpine pasture	5	4	0	1	4483	-0.2	490	677	43	60	731.9
Dry alpine pasture	4	4	0	0	4447	-0.1	488	680	43	60	733.4
Dry alpine pasture	4	3	0	1	3975	3.4	624	792	38	79	697.9
Dry alpine pasture	13	9	2	0	5112	-5.5	375	519	50	45	762.8
Dry alpine pasture	2	2	0	0	5112	-5.5	375	519	50	45	762.8
Dry alpine pasture	5	4	1	0	4711	-1.7	433	612	41	53	747.8
Dry alpine pasture	3	2	0	0	4082	1.9	558	730	38	68	714.5
Temperate coniferous	22	11	4	5	1690	16.1	2120	1074	97	160	529
Dry alpine pasture	5	4	0	0	4082	1.9	558	730	38	68	714.5
Dry alpine pasture	4	2	0	0	4082	1.9	558	730	38	68	714.5
Dry alpine pasture	7	5	2	0	4164	2	569	713	38	68	709.9
Dry alpine pasture	4	3	1	0	4164	2	569	713	38	68	709.9
Dry alpine pasture	4	3	0	1	4164	2	569	713	38	68	709.9
Dry alpine pasture	4	3	0	1	4128	1.6	560	730	38	68	714
Dry alpine pasture	4	3	0	1	3951	2.9	636	745	38	76	700.1
Dry alpine pasture	5	3	0	1	4014	2.6	632	724	39	74	700.5
Dry alpine pasture	5	4	0	1	3862	3	654	748	39	76	695.7
Dry alpine pasture	8	5	0	1	3862	3	654	748	39	76	695.7
Temperate coniferous	20	7	4	8	1690	16.1	2120	1074	97	160	529
Dry alpine pasture	7	6	1	0	3862	3	654	748	39	76	695.7
Dry alpine pasture	7	5	2	0	3862	3	654	748	39	76	695.7
Dry alpine pasture	7	4	1	1	3971	3.6	672	737	39	78	692.3
Dry alpine pasture	4	3	0	1	3971	3.6	672	737	39	78	692.3
Dry alpine pasture	6	5	0	1	3172	7.7	886	873	44	103	652.5
Dry alpine pasture	6	5	0	1	3862	3	654	748	39	76	695.7
Temperate coniferous	6	5	0	1	3466	6.2	820	830	42	94	659.1
Dry alpine pasture	8	7	0	1	4009	3.2	673	727	39	78	690.5
Dry alpine pasture	7	5	0	1	3750	5	750	794	41	87	675.4

Dry alpine pasture	5	4	0	0	3750	5	750	794	41	87	675.4
Pine	6	3	0	3	1484	17.8	2099	1141	100	152	536.9
Dry alpine pasture	5	2	1	2	4156	1.9	625	706	39	72	700.7
Dry alpine pasture	5	4	0	1	4092	1.9	616	702	39	72	702.7
Dry alpine pasture	4	4	0	0	4287	0.1	554	650	40	64	715.5
Dry alpine scrub	17	13	2	2	3319	6.8	847	848	43	98	657.9
Temperate coniferous	21	17	2	2	3466	6.2	820	830	42	94	659.1
Dry alpine scrub	14	12	1	1	3319	6.8	847	848	43	98	657.9
Temperate coniferous	19	14	3	2	3466	6.2	820	830	42	94	659.1
Temperate coniferous	20	16	0	4	3466	6.2	820	830	42	94	659.1
Temperate coniferous	27	18	4	5	3164	7.2	888	866	42	102	647.3
Orchard	26	17	4	4	3054	8.7	1024	899	41	119	629.2
Grassland	27	11	2	12	709	21.6	2387	1424	131	114	605.6
Orchard	15	11	1	3	2906	9.6	1138	924	41	131	620.8
Temperate coniferous	6	5	0	1	2663	10.3	1268	955	41	142	612.2
Temperate coniferous	19	12	4	3	2590	12.1	1746	968	53	176	593.7
Dry alpine pasture	16	10	5	1	2590	12.1	1746	968	53	176	593.7
Temperate coniferous	8	4	2	2	2478	12.1	1763	997	53	177	592.3
Orchard	30	18	4	6	2478	12.1	1763	997	53	177	592.3
Temperate coniferous	23	14	3	3	2923	9.3	1093	913	40	126	622.4
Temperate coniferous	19	13	4	2	2923	9.3	1093	913	40	126	622.4
Temperate coniferous	23	13	6	4	2079	14.4	2089	1052	70	190	570.1
Orchard	25	13	7	3	2151	14.8	2041	1064	72	185	565.1
Sub-tropical pine mixed	23	4	2	14	709	21.6	2387	1424	131	114	605.6
Temperate coniferous	18	10	5	2	2112	14.5	2071	1053	73	187	563.6
Temperate coniferous	24	13	3	6	1368	18.3	1298	1186	59	128	557.2
Temperate coniferous	30	13	8	9	1368	18.3	1298	1186	59	128	557.2
Scrub	18	8	6	4	1974	15.1	1399	1041	76	117	517
Temperate coniferous	17	5	8	4	1361	18.8	1259	1224	59	126	559.9
Temperate coniferous	13	5	2	4	1700	17.3	1470	1144	76	127	540.9
Temperate coniferous	6	0	2	3	1700	17.3	1470	1144	76	127	540.9
Temperate coniferous	6	0	2	3	1700	17.3	1470	1144	76	127	540.9
Temperate coniferous	39	24	6	5	1700	17.3	1470	1144	76	127	540.9
Temperate coniferous	30	15	5	8	1700	17.3	1470	1144	76	127	540.9
Dry alpine pasture	35	21	8	5	2003	14.1	2010	1007	93	141	531.7
Temperate coniferous	24	13	3	6	2174	15.4	1747	1006	86	131	532.1
Temperate coniferous	6	5	1	0	2174	15.4	1747	1006	86	131	532.1
Temperate coniferous	16	8	0	5	2471	11.7	1598	934	73	139	541.9
Temperate coniferous	26	15	2	9	1860	16.5	1460	1078	73	123	540.3
Temperate coniferous	26	11	3	8	1993	17	1357	1064	68	120	540.3
Temperate coniferous	16	10	1	4	2268	13.8	1988	990	91	144	531
Temperate coniferous	19	9	6	3	1993	17	1357	1064	68	120	540.3
Temperate coniferous	20	15	1	4	1860	16.5	1460	1078	73	123	540.3
Temperate coniferous	22	10	6	6	1860	16.5	1460	1078	73	123	540.3
Sub-tropical pine mixed	21	6	4	10	669	22	2299	1430	129	112	614.8
Temperate coniferous	22	11	5	3	3098	8.1	1227	830	52	129	562.1
Temperate coniferous	22	15	3	4	2942	9.2	1301	855	57	132	551.3

Himalayan moist temperate	12	7	1	3	3215	8.1	1230	830	52	129	563.7
Temperate coniferous	19	9	7	3	2620	11.5	1570	910	75	136	537.7
Temperate coniferous	18	11	6	1	2620	11.5	1570	910	75	136	537.7
Temperate coniferous	16	5	6	5	2497	11.1	1511	922	71	136	536.5
Temperate coniferous	21	11	3	6	1612	16.6	1438	1116	73	122	540.5
Pine	12	6	2	4	1612	16.6	1438	1116	73	122	540.5
Temperate coniferous	14	8	1	5	2777	9.3	1310	867	57	132	549.8
Temperate coniferous	19	10	4	4	2329	12.7	1752	945	85	135	526.8
Sub-tropical pine mixed	11	6	2	3	669	22	2299	1430	129	112	614.8
Himalayan moist temperate	34	22	9	2	2521	12	1491	899	81	120	509.3
Temperate coniferous	26	17	2	7	2749	10.5	1388	866	70	129	520.3
Pine	26	16	3	7	2714	11	1420	878	72	128	517.2
Scrub	29	9	4	16	1321	18.6	1100	1248	42	131	562.1
Temperate coniferous	16	12	1	3	1836	16.3	1424	1118	66	129	542
Scrub	25	21	3	0	2295	13.2	1730	993	82	136	531.8
Scrub	18	13	2	1	2261	13.8	1365	995	69	125	518.2
Scrub	29	14	3	12	2613	11.2	1179	912	53	129	521.7
Pine	13	6	1	5	3014	9.3	1269	838	60	136	520.1
Scrub	15	9	1	5	2347	12.1	1417	911	75	126	504
Pine	15	10	2	2	1629	17.2	1511	1118	95	110	529.4
Scrub	12	7	1	4	621	22.3	2220	1459	128	110	624
Temperate coniferous	28	10	8	6	1811	16.1	1287	1062	92	89	515.7
Temperate coniferous	16	7	3	5	2212	13.4	1526	920	99	89	499.6
Temperate coniferous	20	10	3	6	2655	11.1	1473	872	84	117	505.7
Himalayan moist temperate	21	9	6	6	2044	14.3	1421	966	96	87	498.5
Temperate coniferous	16	8	1	6	2703	11.2	1389	873	73	131	504.3
Orchard	25	7	4	11	1655	16.5	1701	1119	73	154	547.9
Himalayan moist temperate	31	12	5	12	1744	16.3	1291	1076	93	90	513.3
Scrub	44	20	5	13	1676	16.9	1279	1091	92	91	523.5
Pine	37	5	9	19	1949	15.1	1391	1003	98	87	506.9
Temperate coniferous	22	14	4	3	2314	12.9	1531	915	88	111	507.4
Scrub	15	4	1	9	687	21.9	2366	1421	129	115	610.1
Scrub	23	13	6	2	2314	12.9	1531	915	88	111	507.4
Temperate coniferous	20	14	4	2	2427	12.2	1513	903	83	119	508.7
Temperate coniferous	22	12	5	3	2570	11.5	1467	892	77	125	512
Temperate coniferous	32	17	6	8	2827	10.6	1380	870	68	131	520.3
Temperate coniferous	25	13	6	3	2120	13.8	1503	947	91	101	504.7
Himalayan moist temperate	31	15	6	8	1836	15.5	1337	1053	81	99	520.1
Scrub	31	25	3	3	1333	18.6	1168	1197	64	111	545.2
Scrub	1	0	0	1	1380	18.7	1170	1226	65	111	551.2
Scrub	1	0	0	1	1380	18.7	1170	1226	65	111	551.2
Scrub	1	1	0	0	1380	18.7	1170	1226	65	111	551.2
Temperate coniferous	18	7	3	7	1462	17.9	2607	1133	113	160	530.5
Scrub	1	1	0	0	1431	18.2	1141	1190	65	104	542.2
Scrub	29	18	2	6	1388	18.3	1149	1234	64	107	544
Dry alpine scrub	21	15	0	5	803	21.4	1483	1380	74	142	592.3
Temperate coniferous	21	7	6	3	1979	12.2	1514	1072	68	137	539.5

Temperate coniferous	18	10	4	4	2175	8.5	1092	1010	41	130	572.2
Temperate coniferous	13	8	2	2	2325	7.9	1089	970	42	130	579
Scrub	1	1	0	0	2325	7.9	1089	970	42	130	579
Cedrus	1	1	0	0	2898	10.3	1148	887	45	131	558
Temperate coniferous	13	9	0	4	2325	7.9	1089	970	42	130	579
Temperate coniferous	18	12	1	5	2325	7.9	1089	970	42	130	579
Temperate coniferous	20	7	4	8	1462	17.9	2607	1133	113	160	530.5
Temperate coniferous	13	6	2	5	2325	7.9	1089	970	42	130	579
Temperate coniferous	18	10	1	6	2325	7.9	1089	970	42	130	579
Temperate coniferous	10	2	3	4	2325	7.9	1089	970	42	130	579
Temperate coniferous	6	1	2	3	2325	7.9	1089	970	42	130	579
Pine	18	9	5	4	2685	8.1	1092	928	42	130	573.5
Scrub	11	4	4	3	2167	13.4	1914	1035	88	141	536
Pine	1	1	0	0	2167	13.4	1914	1035	88	141	536
Scrub	1	0	0	1	2167	13.4	1914	1035	88	141	536
Scrub	1	1	0	0	2050	13.3	1822	996	85	139	537.5
Himalayan moist temperate	39	21	2	15	1462	17.9	2607	1133	113	160	530.5
Temperate coniferous	13	4	3	6	2296	12.3	1558	961	72	135	535.6
Temperate coniferous	25	15	4	6	1756	17	1258	1093	60	123	535.5
Temperate coniferous	35	19	4	12	1756	17	1258	1093	60	123	535.5
Temperate coniferous	19	9	3	4	2351	12.4	1474	935	73	129	521
Scrub	25	16	3	4	2071	13.7	1403	1005	73	125	513.9
Temperate coniferous	32	15	3	9	1428	18.6	1452	1184	77	136	541.5
Pine	31	11	5	9	2431	12.3	1377	920	73	127	504.9
Pine	26	13	1	10	2616	11.3	1354	885	68	132	510.1
Scrub	23	10	1	10	2635	11.4	1372	893	70	131	505.5
Temperate coniferous	16	10	3	2	2539	11.9	1433	887	80	123	500.1
Temperate coniferous	15	8	2	5	1598	17.6	2569	1127	111	161	524.3
Dry alpine pasture	34	19	6	7	4558	0.5	709	657	46	82	677.4
Dry alpine pasture	22	14	2	6	4051	3.6	837	681	41	98	656.1
Dry alpine pasture	28	15	3	10	4908	-2.6	634	553	53	71	692.4
Dry alpine pasture	26	14	4	8	4022	2.6	842	711	44	95	651.7
Himalayan moist temperate	15	7	3	4	1861	16.3	2417	1073	103	165	524.6
Himalayan moist temperate	13	7	3	3	1065	20.2	2781	1255	126	146	561.8
Himalayan moist temperate	19	5	6	8	1059	20.1	2796	1244	125	148	559.9
Scrub	19	4	2	12	922	20.6	2610	1315	122	142	585.2
Pine	32	13	6	11	1058	20.8	1328	1367	108	89	588.5
Temperate coniferous	31	16	4	10	1216	19.8	2376	1225	107	156	579.9
Temperate coniferous	23	11	5	5	2121	14.7	2182	1057	88	167	536.4
Temperate coniferous	26	10	5	10	2164	14.9	2214	1046	89	169	533.8
Temperate coniferous	20	12	3	3	1584	16.6	2178	1148	90	169	538.3
Temperate coniferous	23	13	3	6	2127	14.8	2197	1059	88	169	538.1
Temperate coniferous	29	14	5	8	2127	14.8	2197	1059	88	169	538.1
Temperate coniferous	19	6	5	8	1975	14.5	2135	1081	86	168	539.9
Pine	28	14	6	6	2121	14.7	2182	1057	88	167	536.4
Temperate coniferous	22	17	3	2	2071	13.9	2024	1030	82	162	552.1
Scrub	19	8	5	4	614	22.5	1374	1489	105	95	619.5

Temperate coniferous	19	13	1	5	2322	12.6	1811	984	75	153	577.4
Pine	29	9	8	9	1925	15.7	2323	1093	94	171	526.9
Scrub	13	10	3	0	1517	17.1	2206	1142	94	167	538.7
Temperate coniferous	33	16	8	7	2127	14.8	2197	1059	88	169	538.1
Temperate coniferous	40	18	7	9	2127	14.8	2197	1059	88	169	538.1
Pine	28	8	6	12	1855	16.4	2176	1120	90	167	533.2
Temperate coniferous	33	11	8	12	2164	14.9	2214	1046	89	169	533.8
Temperate coniferous	30	12	8	9	1584	16.6	2178	1148	90	169	538.3
Scrub	30	15	5	9	1071	20.1	2127	1300	98	155	596.1
Pine	18	7	5	6	1080	20	2097	1286	97	154	593.6
Sub-tropical pine mixed	32	11	6	12	592	22.5	1356	1490	106	93	620.4
Pine	16	8	3	5	1332	18.9	2029	1225	92	158	577.3
Temperate coniferous	26	8	5	13	1855	16.4	2176	1120	90	167	533.2
Tropical Moist deciduous	21	7	6	8	720	21.8	2036	1439	132	95	615.5
Pine	23	8	3	11	743	21.8	1238	1429	111	84	607.9
Scrub	16	5	1	9	662	22.4	1255	1497	108	86	627.7
Sub-tropical pine mixed	13	6	3	4	1349	18	1443	1201	103	94	541.5
Sub-tropical pine mixed	16	4	4	8	1332	18.2	1439	1213	102	95	539.1
Scrub	18	7	6	5	1585	17.1	1477	1144	106	95	530.5
Sub-tropical pine mixed	21	9	4	7	1470	17.6	1428	1182	102	94	532
Sub-tropical pine mixed	18	4	4	10	1416	18.1	1416	1204	101	93	538.8
Scrub	21	6	7	7	1623	16.5	1461	1106	105	95	515.6
Sub-tropical pine mixed	16	10	4	2	1500	17.6	1492	1169	104	96	529.4
Sub-tropical pine mixed	22	7	4	10	1609	17.2	1491	1143	105	95	527.8
Scrub	21	4	3	14	2340	12.5	1637	921	89	123	514.6
Sub-tropical pine mixed	29	11	6	11	921	20.6	1497	1391	112	98	587.7
Sub-tropical pine mixed	38	16	6	16	859	20.8	1501	1387	112	100	587.2
Pine	22	15	2	5	1627	16.8	1595	1101	103	107	523.2
Tropical Moist deciduous	35	13	6	14	701	22.1	1369	1448	104	94	610.8
Tropical Sal mixed moist deciduous	17	9	3	5	592	21.7	2142	1471	119	88	536.1
Shorea	13	7	3	3	467	22.3	1995	1526	120	83	550.3
Pine	16	7	4	5	1590	16	1631	1230	101	93	472.6
Pine	19	9	2	8	1590	16	1631	1230	101	93	472.6
Dry deciduous scrub	14	9	1	3	1120	18.2	1654	1383	103	85	487.9
Pine	18	9	2	7	1520	17.4	1572	1256	99	89	487.2
Wet grasslands	16	9	2	4	925	20.3	1882	1446	111	79	492.8
Pine	15	8	2	4	1332	17.8	1893	1338	109	89	484.6
Pine	15	8	4	3	1270	18.4	1906	1364	109	87	487.8
Wet grasslands	19	12	2	5	1919	15.7	1494	1186	96	94	477.4
Pine	12	6	2	3	1363	17.1	1496	1214	103	102	487.2
Pine	12	8	1	3	1184	17.1	1500	1262	103	102	485.8
Pine	14	8	3	3	1764	15.3	1778	1076	111	115	478.1
Pine	15	6	2	7	1692	15.7	1745	1106	112	112	480.9
Himalayan moist temperate	17	7	3	6	1948	14.4	1816	1038	109	120	471.4
Dry deciduous scrub	17	8	3	6	924	20.4	1419	1396	107	107	545.5
Dry deciduous scrub	17	7	1	9	869	19.9	1518	1368	112	107	535.2
Dry deciduous scrub	10	5	2	3	1206	17.5	1710	1219	113	112	506.7

Dry deciduous scrub	11	5	2	4	856	20.3	1280	1407	103	100	542.2
Dry deciduous scrub	9	6	1	2	1179	18.3	1360	1275	104	103	513.4
Dry deciduous scrub	11	3	1	7	932	19.2	1232	1342	102	98	524.7
Dry deciduous scrub	12	7	3	2	1234	18	1420	1261	105	106	512.5
Wet grasslands	14	5	2	7	1451	16.8	1516	1171	103	112	499.5
Dry deciduous scrub	16	8	2	6	1977	14.3	1517	1079	93	113	469.2
Dry deciduous scrub	11	7	1	3	1846	15.3	1533	1142	97	106	468.9
Pine	18	9	3	6	1942	14.5	1523	1112	94	111	467.4
Dry evergreen scrub	12	3	2	7	1023	20	2361	1339	130	134	550.7
Pine	19	11	1	7	992	20.9	2271	1364	127	134	565.8
Pine	16	7	3	6	1157	19.3	2161	1298	122	131	537.8
Dry deciduous scrub	17	8	2	7	1007	20.7	2210	1333	124	135	562.3
Pine	18	8	2	8	965	19.7	2206	1322	124	133	545.7
Degraded forest	20	10	4	6	1234	18.8	2090	1279	120	129	533.2
Degraded forest	13	4	2	6	2237	12.9	1565	976	99	97	449.9
Pine	19	9	2	7	2050	13.4	1805	1009	106	102	448.1
Pine	23	12	5	5	2180	13.3	1771	1000	106	102	448.7
Himalayan moist temperate	26	8	5	12	2441	12.7	1571	962	99	100	447.3
Pine	18	9	1	7	2208	13.3	1733	995	104	101	448.4
Himalayan moist temperate	23	9	1	12	2014	13.5	1926	1022	109	106	448.7
Himalayan moist temperate	19	8	3	7	1860	13.8	2202	1067	114	114	448.5
Pine	23	13	1	8	1560	15.7	1642	1142	107	97	467.4
Pine	28	8	4	13	1772	14	2117	1074	113	112	447.4
Pine	16	10	1	5	1902	13.7	1950	1059	109	108	448
Pine	21	12	1	8	1902	13.7	1950	1059	109	108	448
Pine	19	9	2	8	1843	14	2079	1073	112	113	449
Pine	21	9	3	8	2053	13.7	1951	1036	111	110	454.6
Himalayan moist temperate	19	11	1	7	1633	15.2	1814	1138	111	104	464.6
Himalayan moist temperate	21	9	2	10	1768	14	2017	1068	112	112	453.1
Himalayan moist temperate	17	10	1	6	2051	13.8	1993	1023	111	111	452.4
Himalayan moist temperate	21	10	4	7	2263	13.1	1769	992	105	107	450.3
Pine	21	9	2	9	2051	13.8	1993	1023	111	111	452.4
Pine	19	12	2	5	2263	13.1	1769	992	105	107	450.3
Pine	18	10	2	5	2271	13	1726	983	104	108	449.4
Degraded forest	21	9	3	9	1996	13.5	1920	1022	109	109	451.2
Pine	23	11	2	8	1647	14.6	1851	1109	110	109	457.7
Pine	19	10	2	6	1576	15.2	1750	1141	109	106	465.2
Pine	14	7	3	4	2179	13.7	1476	1059	87	108	477.7
Dry deciduous scrub	22	11	4	7	2008	14.9	1491	1159	93	99	477.1
Himalayan moist temperate	17	9	2	6	2008	14.9	1491	1159	93	99	477.1
Himalayan moist temperate	17	9	1	7	2149	13.7	1402	1090	88	99	476.5
Himalayan moist temperate	21	8	2	10	2478	12.9	1368	1063	85	98	475.8
Himalayan moist temperate	19	9	1	8	2369	12.8	1362	1053	85	98	477.1
Himalayan moist temperate	16	8	2	6	2791	11.3	1323	995	82	97	478.8
Temperate coniferous	15	8	2	5	2533	12.8	1363	1047	85	98	477.9
Pine	15	8	2	5	1944	16.2	1671	1223	100	98	483.3
Degraded forest	19	9	4	5	1944	16.2	1671	1223	100	98	483.3

Dry deciduous scrub	21	9	5	4	2215	14.7	1494	1140	92	99	477.1
Pine	17	9	2	6	1596	16.5	1436	1254	95	91	489.7
Pine	19	11	1	7	1611	16.8	1460	1247	96	92	488.2
Himalayan moist temperate	23	11	3	8	2571	11.1	1556	885	87	129	488
Pine	14	8	1	5	1916	14.4	1785	1077	109	116	467.5
Pine	14	8	1	5	1415	16.9	1465	1194	104	107	493
Himalayan moist temperate	21	11	3	6	1973	13.6	2028	1026	111	108	448.5
Himalayan moist temperate	24	7	1	14	2441	12.7	1571	962	99	100	447.3
Himalayan moist temperate	21	8	1	12	2250	13.1	1681	990	102	101	447
Pine	21	8	1	11	2208	13.3	1733	995	104	101	448.4
Himalayan moist temperate	16	7	2	6	1916	13.7	1999	1036	111	110	450.8
Himalayan moist temperate	22	9	0	13	1642	14.2	1998	1090	112	110	454.6
Himalayan moist temperate	17	8	2	6	2004	13.7	2040	1043	111	112	449.7
Pine	17	8	0	8	1587	14.5	1876	1100	111	106	457.2
Himalayan moist temperate	13	6	1	6	1848	13.7	2030	1050	110	110	449.4
Dry deciduous scrub	19	10	4	5	2057	13.5	1764	1021	104	102	449
Dry deciduous scrub	21	11	2	8	2057	13.5	1764	1021	104	102	449
Pine	19	8	2	9	2239	13.3	1640	999	101	98	450.9
Pine	19	10	1	8	1771	13.9	2205	1071	115	114	449.3
Himalayan moist temperate	15	6	2	7	1792	13.9	1930	1075	110	108	447.2
Himalayan moist temperate	23	12	1	9	1594	14.1	1756	1101	107	104	449.1
Pine	22	12	1	9	1740	14	1940	1079	110	109	445.9
Himalayan moist temperate	18	7	2	9	1740	14	1940	1079	110	109	445.9
Himalayan moist temperate	17	7	1	8	1745	13.9	1913	1079	109	107	447.1
Pine	15	9	1	5	1118	17.9	1726	1262	109	91	486.9
Pine	16	10	3	3	1155	16.8	1654	1234	107	92	474.1
Himalayan moist temperate	12	7	0	5	2096	13.6	1672	1029	102	100	450.4
Himalayan moist temperate	22	10	0	12	2096	13.6	1672	1029	102	100	450.4
Himalayan moist temperate	19	8	0	9	1954	13.8	1773	1047	105	103	451.2
Himalayan moist temperate	15	5	1	8	1954	13.8	1773	1047	105	103	451.2
Dry deciduous scrub	19	11	2	6	1492	14.1	1992	1112	112	107	451.2
Pine	16	9	1	6	1492	14.1	1992	1112	112	107	451.2
Dry deciduous scrub	16	7	1	7	1442	14.3	1867	1139	110	103	452.8
Dry deciduous scrub	15	4	2	9	1424	15.1	1704	1149	108	98	459.3
Himalayan moist temperate	17	8	2	7	2143	12.8	1600	983	100	100	446.4
Degraded forest	12	8	0	4	1882	13.6	2037	1040	111	109	448.7
Tropical Sal mixed moist deciduous	14	5	3	6	593	21.5	2134	1482	119	88	532.3
Himalayan moist temperate	13	8	1	4	2237	12.9	1565	976	99	97	449.9
Pine	22	13	1	8	2237	12.9	1565	976	99	97	449.9
Himalayan moist temperate	21	14	0	7	2237	12.9	1565	976	99	97	449.9
Pine	16	10	1	5	2200	13.2	1738	996	105	101	448.5
Himalayan moist temperate	15	6	3	6	2250	13.1	1681	990	102	101	447
Pine	16	9	0	7	2030	13.5	1896	1024	108	106	451.2
Pine	15	6	1	8	2030	13.5	1896	1024	108	106	451.2
Himalayan moist temperate	16	8	0	8	2030	13.5	1896	1024	108	106	451.2
Pine	12	4	1	7	2030	13.5	1896	1024	108	106	451.2
Himalayan moist temperate	12	6	3	3	2030	13.5	1896	1024	108	106	451.2

Shorea	14	1	3	10	1136	18.1	1775	1291	111	88	491.1
Pine	16	5	3	8	2050	13.4	1805	1009	106	102	448.1
Pine	17	7	1	9	2050	13.4	1805	1009	106	102	448.1
Pine	10	5	1	4	2050	13.4	1805	1009	106	102	448.1
Sub-alpine	11	6	1	4	2429	12.7	1563	961	98	100	447.8
Pine	12	5	2	5	2050	13.4	1805	1009	106	102	448.1
Himalayan moist temperate	15	8	1	6	2014	13.5	1926	1022	109	106	448.7
Himalayan moist temperate	13	5	3	5	1993	13.6	2029	1040	111	110	448.3
Pine	18	6	7	4	2014	13.5	1926	1022	109	106	448.7
Pine	16	6	2	8	1993	13.6	2029	1040	111	110	448.3
Himalayan moist temperate	15	5	1	8	1993	13.6	2029	1040	111	110	448.3
Tropical Sal mixed moist deciduous	11	1	3	7	833	20.7	1991	1410	117	85	519.5
Himalayan moist temperate	17	7	0	10	2441	12.7	1571	962	99	100	447.3
Himalayan moist temperate	15	9	2	4	2441	12.7	1571	962	99	100	447.3
Himalayan moist temperate	15	6	2	6	2441	12.7	1571	962	99	100	447.3
Pine	14	6	1	7	1486	15.3	1651	1146	107	96	462.2
Pine	17	5	3	9	1486	15.3	1651	1146	107	96	462.2
Pine	17	7	2	8	1856	14.3	1780	1059	108	114	465.4
Pine	16	8	1	6	2249	13	1780	988	104	122	456
Pine	24	8	3	12	1742	14.8	1739	1095	109	112	465.3
Himalayan moist temperate	14	7	1	6	1662	15.6	1670	1136	108	108	472.8
Himalayan moist temperate	20	10	1	9	2262	12.7	1769	992	102	122	459.8
Tropical Sal mixed moist deciduous	16	1	2	13	1125	17.8	1771	1305	111	89	488.6
Pine	20	10	4	6	1274	17.3	1540	1229	110	100	496.1
Himalayan moist temperate	9	5	2	2	2143	12.8	1600	983	100	100	446.4
Himalayan moist temperate	13	8	2	3	2143	12.8	1600	983	100	100	446.4
Himalayan moist temperate	12	4	3	4	2016	13.9	2209	1043	115	113	450.5
Himalayan moist temperate	13	6	1	5	2501	12.4	1514	969	88	113	462.9
Pine	12	4	1	5	2442	12.3	1511	975	88	113	463.8
Himalayan moist temperate	17	10	2	4	2295	12.6	1513	995	89	112	460.3
Himalayan moist temperate	19	10	2	6	2199	12.6	1517	1013	89	113	463.3
Himalayan moist temperate	17	7	0	9	2164	13.3	1539	1032	92	114	460.1
Himalayan moist temperate	17	9	2	5	2097	13.6	1546	1041	93	115	463.1
Riverine	16	1	1	14	1020	18	1743	1323	110	87	491.1
Himalayan moist temperate	18	8	2	6	2115	13.5	1708	1015	101	122	465.7
Himalayan moist temperate	13	6	3	3	2455	12.3	1720	965	97	124	465.8
Himalayan moist temperate	20	9	3	7	2357	12.7	1715	983	98	124	467.2
Himalayan moist temperate	16	5	2	7	2145	13.8	1693	1031	101	122	469
Himalayan moist temperate	17	8	2	6	2486	12.1	1711	958	95	125	466.5
Himalayan moist temperate	16	4	1	10	2198	12.9	1701	998	98	123	463.2
Himalayan moist temperate	12	4	1	6	2473	11.7	1656	952	89	126	465.6
Himalayan moist temperate	17	7	5	5	2363	12.7	1642	983	93	123	467.2
Cedrus	15	6	0	9	2473	12.1	1644	963	91	123	467.5
Cedrus	12	4	3	5	2687	10.6	1661	926	85	132	473.4
Shorea	20	2	2	16	980	19.2	1878	1359	112	90	505.6
Himalayan moist temperate	9	5	2	2	2466	11.8	1595	977	85	126	473.3
Himalayan moist temperate	14	4	4	6	2270	13	1549	1022	88	120	467.8

Himalayan moist temperate	15	5	2	7	2139	13.6	1521	1049	90	117	469.9
Himalayan moist temperate	18	7	3	8	2386	12.6	1554	1001	87	119	471.3
Himalayan moist temperate	13	6	1	6	2227	13.1	1538	1024	88	118	467.7
Himalayan moist temperate	16	6	1	7	2542	11.6	1414	886	78	130	496.2
Cedrus	18	10	1	6	2596	12.1	1447	910	81	128	496
Himalayan moist temperate	17	8	5	3	2208	14	1464	980	85	121	500.2
Cedrus	18	7	2	8	1749	15.7	1454	1068	87	122	510.4
Himalayan moist temperate	18	8	1	8	1982	15	1510	1144	95	99	474.8
Tropical Sal mixed moist deciduous	16	2	1	13	1073	18.4	1814	1315	111	94	499.9
Himalayan moist temperate	14	7	1	6	1671	16.2	1635	1224	100	94	483.8
Pine	13	8	1	4	1375	18.8	2202	1381	115	87	482.4
Pine	12	7	0	4	1375	18.8	2202	1381	115	87	482.4
Pine	14	4	4	6	2104	14.2	1471	1083	93	99	464.8
Himalayan moist temperate	15	6	0	9	1892	15.4	1618	1139	100	96	471.1
Pine	12	4	0	8	1863	15.1	1574	1155	98	96	471.3
Himalayan moist temperate	11	3	1	7	1711	16	1664	1191	102	93	476.5
Himalayan moist temperate	8	2	2	4	1827	14.7	1463	1109	95	95	464.2
Himalayan moist temperate	8	3	2	3	1827	14.7	1463	1109	95	95	464.2
Pine	12	4	1	7	1962	13.4	1845	1032	107	105	450.1
Tropical Sal mixed moist deciduous	20	2	4	14	556	21.8	1941	1497	117	89	547
Pine	6	2	1	2	1962	13.4	1845	1032	107	105	450.1
Pine	9	3	2	4	1962	13.4	1845	1032	107	105	450.1
Pine	12	4	2	6	1745	13.9	2183	1064	115	114	451.6
Pine	13	6	2	5	2143	12.8	1600	983	100	100	446.4
Pine	15	6	1	8	2050	13.4	1805	1009	106	102	448.1
Himalayan moist temperate	14	6	2	6	2474	12.2	1493	913	85	126	491.3
Himalayan moist temperate	8	3	1	4	2137	13.8	1593	961	97	118	492
Himalayan moist temperate	23	7	3	13	2607	11.7	1452	896	80	130	494.3
Cedrus	11	4	1	6	2329	13	1552	931	91	122	491
Himalayan moist temperate	12	3	2	7	2474	12.2	1493	913	85	126	491.3
Shorea	15	1	2	12	757	20.8	1737	1448	112	96	536.2
Cedrus	14	4	2	8	2224	12.9	1528	947	89	122	487.9
Shorea	22	2	4	16	553	21.9	1726	1481	114	90	553.7
Tropical Sal mixed moist deciduous	21	6	5	10	440	22.6	2062	1525	122	81	551.2
Shorea	10	2	3	5	915	19.3	1727	1373	111	96	515.2
Tropical Sal mixed moist deciduous	18	6	3	9	575	21.3	2164	1471	117	94	532.4
Tropical Dry deciduous	14	1	3	9	267	24.2	1742	1613	126	53	552.7
Riverine	16	3	3	10	273	24.2	1752	1611	127	53	551.2
Riverine	16	6	1	9	281	24.1	1767	1611	127	54	549
Tropical Sal mixed moist deciduous	27	6	5	15	260	24.2	1752	1616	127	52	553
Tropical Sal mixed moist deciduous	17	7	2	8	302	23.7	1872	1595	127	59	557.8
Tropical Sal mixed moist deciduous	26	7	3	16	235	24.3	1721	1620	127	51	557.8
Riverine	14	4	2	8	441	22.6	2013	1539	122	79	555.4
Tropical Dry deciduous	14	3	3	8	423	22.6	2024	1541	122	79	554.3
Pine	16	5	3	8	1830	15.3	1375	1127	88	113	479.9
Tropical Dry deciduous	12	3	2	7	409	22.9	1972	1551	123	76	559
Tropical Sal mixed moist deciduous	15	6	4	5	444	22.6	2001	1539	122	79	554.5

Tropical Sal mixed moist deciduous	12	4	2	6	474	22.4	2029	1526	121	82	548.5
Mixed plantation	18	5	2	11	210	24.5	1499	1627	125	45	561.7
Shorea	14	4	2	8	219	24.5	1514	1625	124	46	562.2
Mixed plantation	17	5	4	8	207	24.5	1491	1627	124	45	560.7
Shorea	17	5	2	10	302	23.7	1872	1595	127	59	557.8
Mixed plantation	12	4	2	6	216	24.5	1505	1625	124	46	559.9
Tropical Sal mixed moist deciduous	15	5	1	9	363	23.1	1677	1567	119	73	568.4
Tropical Sal mixed moist deciduous	14	4	1	9	466	22.4	1701	1527	117	84	559.5
Mixed plantation	14	5	3	5	301	23.5	1549	1585	119	66	578.8
Mixed plantation	16	3	3	10	316	23.5	1564	1586	120	67	579.8
Pine	20	6	2	12	268	23.8	1344	1603	122	53	590.9
Tectona	10	4	1	4	282	23.7	1405	1597	121	57	588.5
Tropical Sal mixed moist deciduous	19	7	4	8	498	22.8	1859	1542	123	68	533.5
Tropical Sal mixed moist deciduous	17	4	3	10	378	23.5	1855	1573	126	62	542.9
Dry deciduous scrub	10	4	3	3	1666	14.1	1899	1077	110	105	451.2
Dry deciduous scrub	13	5	2	6	1397	15.2	1662	1145	107	97	459.2
Himalayan moist temperate	14	2	3	9	2013	13.6	1900	1031	108	105	449.3
Pine	16	8	2	5	1847	13.7	1916	1042	108	106	448
Pine	17	5	3	9	1775	13.9	1791	1075	106	103	447.2
Pine	15	5	1	9	1693	14	1751	1086	106	103	450.7
Pine	14	3	3	7	1699	14.1	1725	1092	105	101	448.9
Pine	13	5	1	7	1659	14.1	1690	1104	105	101	451.5
Dry deciduous scrub	16	4	1	11	1842	13.9	1722	1072	104	101	449.3
Tropical Sal mixed moist deciduous	18	6	4	8	306	23.7	1890	1593	127	60	559.6
Pine	16	6	2	7	1443	15.2	1443	1167	100	97	460
Dry deciduous scrub	18	8	2	8	1519	14.8	1485	1159	101	99	454.9
Pine	18	7	2	9	1450	15.4	1414	1177	100	95	458.4
Pine	13	7	2	4	1515	15.1	1449	1171	99	95	456
Himalayan moist temperate	19	7	2	9	1687	14.5	1595	1128	101	99	456.8
Dry deciduous scrub	18	7	2	9	1387	17	1808	1281	107	91	473.2
Pine	16	6	2	8	1750	14.9	1588	1152	97	101	471.3
Dry deciduous scrub	21	8	1	11	1033	19.3	1918	1400	111	86	488.7
Dry deciduous scrub	15	7	0	8	1225	18.1	1959	1345	111	89	483.5
Dry deciduous scrub	17	9	2	6	1501	15.8	1669	1211	102	93	470.1

TRI	HF	HNP	AI	SLP
261.88	30	22.49	0.14	5.37
184.16	35	67.21	0.09	1.6
248.1	36	67.21	0.09	5.1
254.24	43	67.21	0.09	0.78
96.78	36	67.21	0.09	1.41
73.86	35	67.21	0.09	1.85
692.46	16	14.35	0.05	5.98
692.46	16	14.35	0.05	5.98
692.46	16	14.35	0.05	5.98
383.41	16	14.35	0.05	2.11
383.41	16	14.35	0.05	2.11
383.41	16	14.35	0.05	2.11
383.41	16	14.35	0.05	2.11
383.41	16	14.35	0.05	2.11
383.41	16	14.35	0.05	2.11
171.76	30	67.21	0.09	3.46
383.41	16	14.35	0.05	2.11
383.41	16	14.35	0.05	2.11
383.41	16	14.35	0.05	2.11
818.19	16	23.84	0.05	3.27
818.19	16	23.84	0.05	3.27
818.19	16	23.84	0.05	3.27
818.19	16	23.84	0.05	3.27
818.19	16	23.84	0.05	3.27
813.83	16	23.84	0.05	16.17
813.83	16	23.84	0.05	16.17
222.45	35	75.54	0.11	1.07
813.83	16	23.84	0.05	16.17
813.83	16	23.84	0.05	16.17
813.83	16	23.84	0.05	16.17
813.83	16	23.84	0.05	16.17
466.06	18	23.84	0.05	2.23
466.06	18	23.84	0.05	2.23
466.06	18	23.84	0.05	2.23
447.01	25	23.84	0.06	5.41
447.01	25	23.84	0.06	5.41
447.01	25	23.84	0.06	5.41
158.78	35	75.54	0.1	3.08
447.01	25	23.84	0.06	5.41
447.01	25	23.84	0.06	5.41
476.03	25	23.84	0.06	5.07
476.03	25	23.84	0.06	5.07
670.88	25	23.84	0.06	4.33
670.88	25	23.84	0.06	4.33
670.88	25	23.84	0.06	4.33
670.88	25	23.84	0.06	4.33

670.88	25	23.84	0.06	4.33
1177.76	18	23.84	0.06	21.4
151.26	36	75.54	0.1	2.82
804.62	18	23.84	0.07	14.34
804.62	18	23.84	0.07	14.34
804.62	18	23.84	0.07	14.34
804.62	18	23.84	0.07	14.34
1164.79	25	9.81	0.07	18.93
1268.3	25	6.03	0.07	25.05
1360.46	31	4.69	0.11	1.97
1360.46	31	4.69	0.11	1.97
1298.7	28	19.48	0.09	29.79
1147.16	31	4.69	0.11	9.61
235.77	36	75.54	0.1	1.05
1147.16	31	4.69	0.11	9.61
1018.22	31	4.69	0.09	22.16
1018.22	31	4.69	0.09	22.16
1014.51	25	4.69	0.09	22.5
783.5	18	4.69	0.08	18.66
947.15	25	4.69	0.09	22.12
1055.73	18	4.69	0.09	22.45
945.92	11	4.77	0.07	24.82
945.92	11	4.77	0.07	24.82
617.91	36	15.09	0.09	5.48
644.81	23	22.49	0.15	12.23
83.77	70	75.54	0.13	0.45
617.91	36	15.09	0.09	5.48
617.91	36	15.09	0.09	5.48
617.91	36	15.09	0.09	5.48
617.91	36	15.09	0.09	5.48
617.91	36	15.09	0.09	5.48
617.91	36	15.09	0.09	5.48
677.02	36	15.09	0.09	21.93
836.37	36	15.09	0.09	21.79
677.02	36	15.09	0.09	21.93
677.02	36	15.09	0.09	21.93
124.83	70	75.54	0.12	2.6
677.02	36	15.09	0.09	21.93
677.02	36	15.09	0.09	21.93
678	25	15.09	0.09	20.15
836.37	36	15.09	0.09	21.79
678	25	15.09	0.09	20.15
678	25	15.09	0.09	20.15
678	25	15.09	0.09	20.15
1067.47	28	5.85	0.11	22.27
1693.19	28	5.85	0.11	19.4
986.71	31	9.18	0.13	4.64

180.07	50	65.74	0.13	3.49
1046.71	31	6.03	0.12	17.92
1062.16	31	9.18	0.13	12.28
1314.18	25	6.03	0.07	16.69
1211.93	25	9.18	0.08	24.58
804.29	25	6.03	0.1	8.1
804.29	25	6.03	0.1	8.1
804.29	25	6.03	0.1	8.1
1010.11	25	6.03	0.09	36.45
1129.91	25	6.03	0.09	38.84
1379.43	25	6.03	0.08	29.4
212.08	62	62.34	0.14	2.75
1379.43	25	6.03	0.08	29.4
959.07	31	9.18	0.1	18.82
959.07	31	9.18	0.1	18.82
928.37	31	9.18	0.12	8.79
928.37	31	9.18	0.12	8.79
1024.02	31	9.18	0.09	18.57
998.8	31	9.18	0.1	12.66
849.58	18	4.69	0.08	19.97
1057.21	18	4.69	0.07	24.55
124.95	41	65.74	0.12	1.85
940.64	18	3.72	0.07	19.11
991.91	18	3.72	0.08	18.02
817.33	25	3.72	0.09	10.37
817.33	25	3.72	0.09	10.37
817.33	25	3.72	0.09	10.37
817.33	25	3.72	0.09	10.37
817.33	25	3.72	0.09	10.37
923.05	18	3.72	0.08	21.87
1032.17	18	3.72	0.07	23.18
112.7	23	65.74	0.1	1.37
1350.44	18	3.72	0.08	13.98
681.12	18	3.72	0.07	16.34
1051.47	18	3.72	0.07	20.3
693.89	25	3.72	0.1	11.66
1036.13	25	3.72	0.09	20.94
693.89	25	3.72	0.1	11.66
1036.13	25	3.72	0.09	20.94
1036.13	25	3.72	0.09	20.94
998.68	25	3.72	0.1	4.67
721.96	25	4.69	0.09	17.12
121.45	36	65.74	0.1	1.71
332.31	11	9.81	0.06	6.1
648.14	25	4.69	0.1	8.66
1055.99	25	4.69	0.08	26.95
1567.56	25	4.69	0.08	23.97

1055.99	25	4.69	0.08	26.95
648.31	25	4.69	0.1	11.64
775.83	25	3.72	0.1	16.13
775.83	25	3.72	0.1	16.13
968.18	25	3.72	0.09	22.85
857.08	25	3.72	0.09	19.52
267.02	36	75.54	0.1	4.84
968.18	25	3.72	0.09	22.85
968.18	25	3.72	0.09	22.85
985.62	11	5.25	0.07	22.98
756.15	18	5.25	0.08	15.54
1119.47	25	15.09	0.09	26.28
733.71	25	5.25	0.09	5.52
733.71	25	5.25	0.09	5.52
733.71	25	5.25	0.09	5.52
1140.64	25	15.09	0.09	23.75
745.32	30	15.09	0.09	16.47
218.03	40	70.31	0.14	2.07
648.42	30	15.09	0.1	10.58
1232.93	30	15.09	0.1	17.64
1123.76	25	15.09	0.09	26.08
1033.47	25	15.09	0.1	11.19
1013.29	18	15.09	0.09	26.09
1013.29	18	15.09	0.09	26.09
772.54	30	25.61	0.1	12.22
799.97	28	12.82	0.14	16.73
1292.89	23	12.82	0.16	29.5
662.08	18	15.09	0.08	10.98
77	40	70.31	0.15	1.11
887.14	23	12.82	0.13	14.4
1154.57	36	42.67	0.15	13.31
1154.57	36	42.67	0.15	13.31
1154.57	36	42.67	0.15	13.31
1119.62	23	42.67	0.19	23.64
1273.3	23	12.82	0.19	23.61
1009.81	30	42.67	0.12	24.53
1202.14	36	42.67	0.14	7.58
1202.14	36	42.67	0.14	7.58
433.84	30	13.5	0.18	12.7
105.9	73	41.06	0.2	2.91
1213.01	30	42.67	0.13	10.24
1169.08	34	13.48	0.1	18.15
1023.21	34	8.95	0.11	1.39
1312.6	34	8.95	0.1	26.91
1312.6	34	8.95	0.1	26.91
949.04	30	5.44	0.11	5.21
949.04	30	5.44	0.11	5.21

949.04	30	5.44	0.11	5.21
949.04	30	5.44	0.11	5.21
949.04	30	5.44	0.11	5.21
134.73	30	41.06	0.2	3.35
1202.14	36	42.67	0.14	7.58
1202.14	36	42.67	0.14	7.58
866.06	30	5.44	0.12	11.4
866.06	30	5.44	0.12	11.4
866.06	30	5.44	0.12	11.4
785.51	28	17.72	0.16	18.86
870.4	30	5.44	0.12	11.4
870.4	30	5.44	0.12	11.4
858.25	34	9.56	0.13	15.2
705.68	21	34.91	0.12	14.61
990.85	23	41.06	0.2	18.63
1046.39	43	12.23	0.12	22.83
904.17	16	4.58	0.07	19.47
747.47	25	5.25	0.08	17.76
1046.39	43	12.23	0.12	22.83
760.41	30	12.23	0.12	10.14
836.82	30	10.36	0.09	23.63
1087.91	30	5.44	0.1	4
1087.91	30	5.44	0.1	4
876.5	30	5.44	0.11	20.02
876.5	30	5.44	0.11	20.02
816.97	30	41.06	0.2	13.37
876.5	30	5.44	0.11	20.02
876.5	30	5.44	0.11	20.02
876.5	30	5.44	0.11	20.02
867.7	30	5.44	0.11	20.02
867.7	30	5.44	0.11	20.02
936.48	30	5.44	0.11	15.81
936.48	30	5.44	0.11	15.81
936.48	30	5.44	0.11	15.81
936.48	30	5.44	0.11	15.81
936.48	30	5.44	0.11	15.81
1011.14	25	9.18	0.08	15.05
936.48	30	5.44	0.11	15.81
825.1	30	5.44	0.1	4.38
825.1	30	5.44	0.1	4.38
1432.04	30	5.44	0.12	33.86
965.95	30	5.44	0.1	10.17
1177.38	30	5.44	0.1	26.42
1177.38	30	5.44	0.1	26.42
1087.91	30	5.44	0.1	4
1177.38	30	5.44	0.1	26.42
599.64	50	41.65	0.17	13.24

1177.38	30	5.44	0.1	26.42
1177.38	30	5.44	0.1	26.42
1177.38	30	5.44	0.1	26.42
1177.38	30	5.44	0.1	26.42
1087.91	30	5.44	0.1	4
1087.91	30	5.44	0.1	4
1087.91	30	5.44	0.1	4
1087.91	30	5.44	0.1	4
857.39	30	5.44	0.09	2.16
892.09	30	5.44	0.09	6.63
615.03	50	41.65	0.19	13.82
899.54	30	8.67	0.09	8.82
899.54	30	8.67	0.09	8.82
767.99	28	13.48	0.1	18.69
916.33	40	8.67	0.08	27.94
875.35	28	8.67	0.08	25.72
734	40	8.67	0.08	16.03
856.83	36	8.67	0.07	11.54
831.48	31	8.67	0.07	10.76
1307.96	31	8.67	0.07	2.71
615.03	50	41.65	0.19	13.82
451.75	42	8.67	0.07	9.91
1209	29	8.67	0.08	22.78
1062.93	25	8.67	0.07	12.18
1050.48	25	8.67	0.1	18.61
1050.48	25	8.67	0.1	18.61
910.74	25	8.67	0.09	6.98
846.91	25	8.67	0.09	6.98
802.33	25	8.67	0.08	3.29
835.2	31	8.67	0.07	9.88
892.83	35	8.67	0.07	16.68
702.86	30	34.91	0.2	15.42
495.21	36	8.34	0.07	18.94
903.37	36	10.36	0.07	12.81
771.88	36	10.36	0.07	10.64
1110.45	26	10.36	0.07	7.13
665.38	26	4.58	0.07	13.72
625.94	23	2.1	0.08	1.77
1104.79	23	2.1	0.08	5.74
769.07	23	2.1	0.09	24.27
417.15	23	2.1	0.09	7.35
796.44	30	41.06	0.19	11.98
742.1	23	2.1	0.08	20.45
644.64	23	2.1	0.08	22.89
760.71	23	4.38	0.08	19.87
773.99	23	2.1	0.08	10.71
453.7	16	4.38	0.07	10.19

584.07	16	4.38	0.07	11.17
362.21	16	4.38	0.07	13.2
362.21	16	4.38	0.07	13.2
362.21	16	4.38	0.07	13.2
603.62	23	22.49	0.15	8.12
664.36	30	34.91	0.19	14.75
362.21	16	4.38	0.07	13.2
638.75	16	4.38	0.07	13.92
396.03	16	4.38	0.07	2.55
284.89	16	4.38	0.07	9.01
396.03	16	4.38	0.07	2.55
284.89	16	4.38	0.07	9.01
284.89	16	4.38	0.07	9.01
358.07	16	4.38	0.07	10.87
362.21	16	4.38	0.07	13.2
362.21	16	4.38	0.07	13.2
796.44	30	41.06	0.19	11.98
307.78	16	2.1	0.07	7.78
307.78	16	2.1	0.07	7.78
307.78	16	2.1	0.07	7.78
384.07	16	2.1	0.07	8.29
834.59	16	2.1	0.08	12.48
791.7	18	5.25	0.07	17.51
791.7	18	5.25	0.07	17.51
802.55	18	5.25	0.07	19.23
811.08	18	5.25	0.08	9.96
816.97	30	41.06	0.2	13.37
811.08	18	5.25	0.08	9.96
811.08	18	5.25	0.08	9.96
710.67	18	5.25	0.08	22.57
710.67	18	5.25	0.08	22.57
710.67	18	5.25	0.08	22.57
935.75	25	5.25	0.08	18.06
788.65	18	5.25	0.08	3.75
617.91	36	15.09	0.09	5.48
680.48	36	25.61	0.09	14.16
680.48	36	25.61	0.09	14.16
816.97	30	41.06	0.2	13.37
680.48	36	25.61	0.09	14.16
680.48	36	25.61	0.09	14.16
891.9	36	15.09	0.09	19.36
891.9	36	15.09	0.09	19.36
676.97	25	15.09	0.1	3.02
680.48	36	25.61	0.09	14.16
899.21	36	25.61	0.1	21.22
641.54	36	25.61	0.09	18.59
838.05	36	25.61	0.09	13.29

838.05	36	25.61	0.09	13.29
459.3	43	41.06	0.18	9.39
802.61	30	15.09	0.09	21.51
920.06	30	15.09	0.09	15.55
651.23	18	15.09	0.09	11.9
961.86	36	25.61	0.1	21.91
899.21	36	25.61	0.1	21.22
961.86	36	25.61	0.1	21.91
899.21	36	25.61	0.1	21.22
899.21	36	25.61	0.1	21.22
704.37	36	25.61	0.11	17.13
771.65	36	25.61	0.12	14.45
265.66	34	70.31	0.16	7.91
666.22	36	25.61	0.12	9.77
617.75	36	25.61	0.15	15.17
700.18	30	25.61	0.16	14.99
700.18	30	25.61	0.16	14.99
671.22	36	25.61	0.18	13.48
671.22	36	25.61	0.18	13.48
641.15	30	25.61	0.12	14.61
641.15	30	25.61	0.12	14.61
929.49	36	15.2	0.2	16.43
784.33	36	15.2	0.19	15.5
265.66	34	70.31	0.16	7.91
989.69	30	15.2	0.2	23.61
880.23	46	12.82	0.11	11.24
880.23	46	12.82	0.11	11.24
354.32	34	17.22	0.13	5.89
1208.21	46	12.82	0.1	20.3
1269.75	34	22.49	0.13	21.4
1269.75	34	22.49	0.13	21.4
1269.75	34	22.49	0.13	21.4
1269.75	34	22.49	0.13	21.4
1269.75	34	22.49	0.13	21.4
1149.58	21	9.56	0.2	2.3
718.61	21	9.56	0.2	10.42
718.61	21	9.56	0.2	10.42
1025.28	21	9.56	0.18	17.98
775.22	21	9.56	0.15	18.59
679.35	34	9.56	0.16	12.01
1036.71	21	9.56	0.2	26.88
679.35	34	9.56	0.16	12.01
775.22	21	9.56	0.15	18.59
775.22	21	9.56	0.15	18.59
234.26	34	70.31	0.16	4.93
862.36	28	9.56	0.15	8.36
510.55	28	9.56	0.15	8.03

901.47	28	9.56	0.15	8.36
733.11	28	9.56	0.17	14.78
733.11	28	9.56	0.17	14.78
766.23	28	9.56	0.18	15.04
975.97	34	9.56	0.13	3.97
975.97	34	9.56	0.13	3.97
845.62	28	9.56	0.16	20.97
626.47	30	9.56	0.19	18.31
234.26	34	70.31	0.16	4.93
754.8	28	29.15	0.17	13.41
949.66	28	29.15	0.16	13.41
651.62	34	19.65	0.16	2.95
688.8	65	9.61	0.09	8.62
1092.97	60	9.61	0.13	21.37
981.13	67	11.62	0.18	22.13
659.76	40	14.86	0.14	14.62
717.48	21	14.86	0.13	15.64
627.25	28	17.22	0.15	14.78
889.18	21	17.22	0.16	9.72
488.09	36	22.49	0.14	7.75
191.29	53	48.5	0.15	1.99
374.44	40	53.71	0.12	5.89
532.04	65	53.71	0.17	8.94
678.8	30	29.15	0.17	4.83
638.69	23	29.15	0.15	14.11
618.51	23	17.22	0.16	2.21
657.15	40	17.72	0.15	12.24
309.29	34	53.71	0.12	1.84
543.98	40	53.71	0.12	13.54
527.39	40	53.71	0.14	7.69
696.85	28	29.15	0.17	7.65
102.5	40	70.31	0.17	1.97
696.85	28	29.15	0.17	7.65
775.53	34	29.15	0.17	19.43
591.52	34	29.15	0.16	17.57
847.7	34	19.65	0.16	13.26
569.86	46	29.15	0.16	10.34
667.79	46	19.65	0.12	8.5
887.05	46	19.65	0.1	8.85
767.91	46	19.65	0.1	17.04
767.91	46	19.65	0.1	17.04
767.91	46	19.65	0.1	17.04
456.92	65	48.5	0.23	11.08
802.14	46	19.65	0.1	15.65
758.41	46	19.65	0.09	9.88
921.26	46	19.65	0.1	2.25
806.36	28	9.61	0.17	6.05

733.24	28	19.48	0.21	6.37
793.37	28	19.48	0.17	14.44
793.37	28	19.48	0.17	14.44
972.56	28	9.61	0.12	20.87
793.37	28	19.48	0.17	14.44
793.37	28	19.48	0.17	14.44
456.92	65	48.5	0.23	11.08
793.37	28	19.48	0.17	14.44
793.37	28	19.48	0.17	14.44
793.37	28	19.48	0.17	14.44
793.37	28	19.48	0.17	14.44
982.32	28	19.48	0.14	17.8
934.46	28	9.61	0.21	12.35
934.46	28	9.61	0.21	12.35
934.46	28	9.61	0.21	12.35
717.29	32	9.61	0.19	23.25
456.92	65	48.5	0.23	11.08
1398.68	60	19.48	0.17	25.07
970.72	28	14.47	0.13	18.3
970.72	28	14.47	0.13	18.3
737.98	40	14.47	0.16	9.41
721.8	21	14.86	0.14	17.48
1121	40	14.86	0.12	12.53
668.84	21	14.86	0.15	14.31
519.86	28	17.22	0.15	8.25
770.68	21	17.22	0.15	9.61
609.74	36	29.15	0.16	8.88
576.81	59	48.5	0.23	11.08
1187	23	30.23	0.11	21.39
1314.76	30	30.23	0.11	33.82
634.37	23	30.23	0.11	10.11
1052.53	36	30.23	0.12	12.7
520.57	54	48.5	0.22	11.17
170.44	36	48.5	0.22	3.42
146.73	58	48.5	0.22	1.21
295.86	21	28.48	0.2	4.84
676.12	71	51.78	0.1	13.69
728.21	34	28.48	0.19	11.31
469.99	28	25.07	0.21	10.88
561.41	28	25.07	0.2	10.88
744.65	40	25.07	0.19	8.01
684.29	28	25.07	0.21	6.35
684.29	28	25.07	0.21	6.35
598.42	40	25.07	0.21	8.27
469.99	28	25.07	0.21	10.88
569.84	28	25.07	0.2	10.83
474.47	50	51.78	0.09	3.73

575.88	28	25.07	0.18	17.17
583.61	54	32.98	0.21	7.09
660.51	40	25.07	0.19	11.19
684.29	28	25.07	0.21	6.35
684.29	28	25.07	0.21	6.35
599.37	54	32.98	0.19	7.04
561.41	28	25.07	0.2	10.88
744.65	40	25.07	0.19	8.01
503.1	46	25.07	0.16	19.11
388.09	34	25.07	0.16	11.45
257.43	50	51.78	0.09	2.92
760.17	46	25.07	0.17	12.11
599.37	54	32.98	0.19	7.04
134.35	30	63.94	0.14	3.41
138.69	43	75.54	0.09	3.04
327.32	50	74.64	0.08	7.6
458.48	43	47.73	0.12	7.3
276.95	43	47.73	0.12	4.02
378.7	23	130.51	0.13	1.46
455.26	23	47.73	0.12	9.85
536.64	23	47.73	0.12	9.29
375.85	43	47.73	0.13	2.99
480.19	30	47.73	0.13	8.85
494.23	43	47.73	0.13	9.85
502.98	23	13.5	0.18	7.84
415.37	78	130.51	0.11	2.88
727.9	65	130.51	0.11	6.26
919.68	30	51.78	0.14	10.45
94.21	66	51.78	0.09	2.15
489.75	35	40.42	0.14	4.02
282.55	43	31.04	0.13	5.31
609.15	36	39.34	0.13	12.75
609.15	36	39.34	0.13	12.75
713.01	30	22.36	0.12	15.08
1118.17	43	22.36	0.12	25.74
583.98	23	39.34	0.13	5.39
380.43	28	39.34	0.14	8.07
760.41	28	26.76	0.14	7.72
639.52	34	26.76	0.13	11.6
489.67	23	24.63	0.12	4.26
425.14	23	24.63	0.12	9.26
436.02	30	33.78	0.17	9.71
451.83	30	33.78	0.16	6.76
523.9	30	33.78	0.18	7.94
439.56	36	23.94	0.1	13.08
685.02	36	23.94	0.11	8.15
906.43	36	23.94	0.14	23.39

637.14	65	36.55	0.09	10.96
668.53	43	27.33	0.11	14.25
811.43	48	27.33	0.09	10.42
668.29	43	27.33	0.11	10.66
736.81	30	27.33	0.13	13.03
649.83	34	21.3	0.14	15.37
389.02	21	23.02	0.13	6.72
311.49	34	21.3	0.14	7.14
608.75	43	21.68	0.18	2.98
510.88	43	21.68	0.17	5.92
631.43	43	21.68	0.17	9.88
702.78	35	21.68	0.17	2.19
822.57	30	21.68	0.17	0.98
483.11	43	21.68	0.17	5.01
725.98	70	31.04	0.16	7.81
643.62	35	31.04	0.18	9.22
692.81	51	31.04	0.17	11.25
619.82	35	31.04	0.16	7.23
751.64	35	31.04	0.17	15.28
863.3	35	31.04	0.19	20.19
651.35	43	31.04	0.21	7.62
558.76	43	31.04	0.15	11.57
548.34	43	40.42	0.2	9.38
926.74	46	31.04	0.2	16.85
926.74	46	31.04	0.2	16.85
825.49	60	40.42	0.19	5.46
896.41	23	43.53	0.19	9.88
717.65	23	43.53	0.16	10.53
857.18	36	43.53	0.19	12.61
690.85	36	31.04	0.19	16.48
629.75	36	31.04	0.18	8.48
690.85	36	31.04	0.19	16.48
629.75	36	31.04	0.18	8.48
475.91	23	31.04	0.17	7.4
673.82	23	31.04	0.19	15.82
481.88	43	43.53	0.17	6.85
346.5	43	43.53	0.15	6.38
872.85	34	56.47	0.14	17.31
1088.35	40	8	0.13	19.98
1088.35	40	8	0.13	19.98
1049.11	40	8	0.13	23.43
1180.34	28	8	0.13	19.86
1067.54	28	8	0.13	22.6
1146.32	28	8	0.13	14.22
1058.79	28	8	0.13	23.81
1113.26	21	8	0.13	11.27
1113.26	21	8	0.13	11.27

1150.56	34	8	0.13	8.07
527.84	30	26.76	0.11	11.73
642.2	43	26.76	0.11	14.69
618.36	36	21.68	0.18	16.46
1059.84	36	24.95	0.16	5.04
554.24	23	36.55	0.12	10.94
503.1	51	31.04	0.19	9.42
619.82	35	31.04	0.16	7.23
590.21	43	31.04	0.17	12.59
751.64	35	31.04	0.17	15.28
749.15	30	31.04	0.19	17.5
545.73	43	31.04	0.19	14.01
929.61	30	31.04	0.2	16.7
862.47	30	31.04	0.18	21.27
798.31	43	40.42	0.2	19.01
698.03	43	40.42	0.18	10.11
698.03	43	40.42	0.18	10.11
973.5	43	40.42	0.16	2.15
623.77	43	40.42	0.21	13.77
699.51	23	40.42	0.19	15.41
611.68	36	40.42	0.16	11.6
657.03	23	40.42	0.18	13.73
657.03	23	40.42	0.18	13.73
519.08	36	40.42	0.18	12.19
1000.88	36	40.42	0.13	4.03
1201.58	36	40.42	0.13	20
509.93	23	40.42	0.16	10.81
509.93	23	40.42	0.16	10.81
453.54	23	40.42	0.17	10.81
453.54	23	40.42	0.17	10.81
479.66	36	40.42	0.17	5.2
479.66	36	40.42	0.17	5.2
519.22	36	40.42	0.15	9.67
350.12	46	40.42	0.15	4.83
555.92	35	31.04	0.17	11.17
772.81	51	31.04	0.2	14.42
381.36	65	40.42	0.14	0.6
725.98	70	31.04	0.16	7.81
725.98	70	31.04	0.16	7.81
725.98	70	31.04	0.16	7.81
539.89	48	31.04	0.18	13.75
590.21	43	31.04	0.17	12.59
691.07	43	31.04	0.19	17.02
691.07	43	31.04	0.19	17.02
691.07	43	31.04	0.19	17.02
691.07	43	31.04	0.19	17.02
691.07	43	31.04	0.19	17.02

539.11	23	37.85	0.14	10.5
643.62	35	31.04	0.18	9.22
643.62	35	31.04	0.18	9.22
643.62	35	31.04	0.18	9.22
776.64	51	31.04	0.16	10.86
643.62	35	31.04	0.18	9.22
863.3	35	31.04	0.19	20.19
1038.94	35	31.04	0.2	22.03
863.3	35	31.04	0.19	20.19
1038.94	35	31.04	0.2	22.03
1038.94	35	31.04	0.2	22.03
523.59	23	37.85	0.14	7.43
619.82	35	31.04	0.16	7.23
619.82	35	31.04	0.16	7.23
619.82	35	31.04	0.16	7.23
569.14	46	40.42	0.15	12.19
569.14	46	40.42	0.15	12.19
681.37	23	24.95	0.17	15.44
601.95	23	18.77	0.18	8.9
801.9	23	18.77	0.16	10.76
627.81	30	18.77	0.15	13.89
593.63	23	18.77	0.18	9.36
512.6	45	37.85	0.14	11.88
425.36	23	24.95	0.13	11.54
555.92	35	31.04	0.17	11.17
555.92	35	31.04	0.17	11.17
491.59	43	31.04	0.2	13.75
506.18	23	23.02	0.16	5.43
622.03	23	23.02	0.15	9.12
464.13	23	23.02	0.15	8.05
400.79	23	23.02	0.15	11.5
448.8	23	23.02	0.15	12.6
331.46	23	23.02	0.15	5.82
672.35	23	37.85	0.13	5.78
527.25	21	36.55	0.17	11.56
800.4	21	36.55	0.18	1.24
557.21	21	36.55	0.17	3.59
577.22	21	36.55	0.16	12.63
876.74	21	36.55	0.18	3.59
520.81	21	36.55	0.17	4.52
318.53	21	20.82	0.17	3.43
411.56	21	20.82	0.17	8.08
301.82	21	20.82	0.17	8.06
427.4	21	20.82	0.18	6.84
700.84	65	40.42	0.14	14.79
630.29	28	16.76	0.16	9.79
739.53	21	16.76	0.15	16.88

769.7	34	16.76	0.15	16.56
626.42	21	16.76	0.16	10.64
672.1	21	16.76	0.15	7.42
693.46	21	15.34	0.16	6.46
949.99	34	15.34	0.16	12.76
730.5	21	15.34	0.15	10.97
1149.97	21	15.34	0.14	17.94
351.79	21	18.52	0.13	6.53
492.97	23	31.04	0.14	11.02
467	40	8	0.13	13.66
585.31	46	8	0.16	10.79
585.31	46	8	0.16	10.79
768.01	43	39.34	0.14	4.72
612.55	30	39.34	0.14	11.01
643.63	30	39.34	0.14	12.54
542.42	23	39.34	0.13	8.64
291.6	53	22.36	0.13	0.71
291.6	53	22.36	0.13	0.71
502.21	43	31.04	0.19	4.12
90.11	23	31.04	0.13	2.12
502.21	43	31.04	0.19	4.12
502.21	43	31.04	0.19	4.12
996.24	30	31.04	0.21	17.02
555.92	35	31.04	0.17	11.17
643.62	35	31.04	0.18	9.22
652.78	28	15.34	0.16	12
742.05	21	15.34	0.17	14.94
696.96	28	15.34	0.16	11.68
755.05	28	15.34	0.16	17.72
652.78	28	15.34	0.16	12
356.93	30	24.61	0.12	7.53
653.26	21	15.34	0.16	13.21
301	30	24.61	0.12	6.42
351.56	30	31.04	0.14	5.63
472.37	23	24.61	0.13	3.29
404.23	43	31.04	0.15	6.91
14	28	17.46	0.11	0.27
20.37	34	17.46	0.11	0.57
28.9	34	17.46	0.11	0.76
21.75	28	25.72	0.11	0.57
23.52	28	37.85	0.12	0.34
33.18	21	25.72	0.11	0.6
118.87	30	31.04	0.13	2.64
183.97	30	31.04	0.13	3.21
737.66	28	14.23	0.12	15.41
66.37	30	31.04	0.13	1.63
83.95	46	31.04	0.13	2.02

102.83	46	31.04	0.13	2.51
14.14	34	51.51	0.09	0.31
12.88	34	51.51	0.09	0.26
9.17	28	51.51	0.09	0.14
23.52	28	37.85	0.12	0.34
10.15	34	51.51	0.09	0.21
44.24	43	25.35	0.11	1.06
61.07	35	25.35	0.11	0.95
25.12	23	25.35	0.1	0.58
26.21	43	25.35	0.1	0.41
19.9	23	47.5	0.08	0.3
26.12	23	47.5	0.09	0.45
275.93	28	17.46	0.12	6.09
344.63	28	17.46	0.12	2.31
441.1	43	40.42	0.2	8.58
505.02	43	40.42	0.15	2.27
603.55	23	40.42	0.18	12.21
517.12	23	40.42	0.19	13
518.59	30	40.42	0.17	12.01
422.81	43	40.42	0.16	4.96
421.78	30	26.42	0.16	5.82
249.19	43	26.42	0.15	6.17
390.32	30	26.42	0.16	10.78
21.95	28	37.85	0.12	0.7
310.77	30	33.41	0.12	8.5
341.21	30	33.41	0.13	3.31
239.44	30	33.41	0.12	2.88
385.95	23	25.59	0.12	7.67
541.9	23	25.59	0.14	12.84
443.01	23	27.56	0.14	7.61
654.12	36	27.56	0.14	12.17
725.04	23	18.52	0.14	6.32
808.16	21	18.52	0.15	20.36
786.45	36	39.34	0.14	10.94
